# Food allergy severity across the world: A World Allergy Organization international survey

**DOI:** 10.1016/j.waojou.2025.101123

**Published:** 2025-11-12

**Authors:** Stefania Arasi, Mário Morais-Almeida, Bryan L. Martin, Gary Wing-Kin Wong, Ignacio J. Ansotegui, Motohiro Ebisawa, Adnan Custovic, Alexandra Santos, Anna Nowak-Wegrzyn, Andrew Stoddart, Antoine Deschildre, Antonella Cianferoni, Antonella Muraro, Audrey DunnGalvin, Brian Vickery, Carina Venter, Carla Jones, Carmen Mazzuca, Christopher Warren, Daniel Munblit, David B. Peden, David Fleischer, Elham Hossny, Graham Roberts, Hania Szajewska, Helen A. Brough, James L. Sublett, Jonathan A. Bernstein, José Antonio Ortega-Martell, Liang-Lu Wang, Luciana Kase Tanno, Luis Caraballo, Manana Chikhladze, Marcia Podestà, Marcus S. Shaker, María Antonieta Guzmán Meléndez, Maria Said, Marta Vazquez-Ortiz, Martin Bozzola, Matthew Greenhawt, Michael Levin, Montserrat Álvaro Lozano, Nikolaos G. Papadopoulos, Olga Patricia Monge Ortega, Paul J. Turner, Paula Kauppi, Pedro Giavina-Bianchi, Philip W. Rouadi, Philippe Bégin, Philippe Eigenmann, R. Maximiliano Gómez, Robert J. Boyle, Ruchi S. Gupta, Sayantani B. Sindher, R. Sharon Chinthrajah, Tonya Winders, Ulugbek Nurmatov, Victoria Cardona, Yoon-Seok Chang, Jennifer Gerdts, Renata Rapillo, Michele Miraglia Del Guidice, Vincenzo Patella, Alessandro Fiocchi, Lamia Dahdah

**Affiliations:** a Allergy Diseases Research Area, Pediatric Allergology Unit, Bambino Gesù Children's Hospital IRCCS, Rome, Italy; b Allergy Center, CUF Descobertas Hospital, Lisbon, Portuguese Republic; c Medicine and Pediatrics, The Ohio State University, Columbus, Ohio, United States; d The Chinese University of Hong Kong, Hong Kong Special Administrative Region of China; e Hospital Quironsalúd Bizkaia, Bilbao, Spain; f Sagamihara National Hospital, Sagamihara, Japan; g Department of Paediatrics, Imperial College London, London, United Kingdom; h King’s College London, and Evelina London Children’s Hospital, Guy’s and St. Thomas’ Hospital, London, United Kingdom; i New York University School of Medicine, Langone Health, New York, United States; j University of Edinburgh, Edinburgh, United Kingdom; k Hospital Center University of Lille, Hôpital Jeanne de Flandre, Lille, France; l The Children’s Hospital of Philadelphia, The University of Pennsylvania, Philadelphia, Pennsylvania, United States; m Food Allergy Centre Department of Woman and Child Health, Padua University Hospital, Padua, Italy; n Applied Psychology and Paediatrics and Child Health, University College Cork, Cork, Ireland; o Department of Pediatrics, Emory University, Atlanta, Georgia, United States; p Section of Allergy and Immunology, Children's Hospital Colorado, University of Colorado School of Medicine, Aurora, Colorado, United States; q Allergy UK, London, United Kingdom; r Allergy Diseases Research Area, Pediatric Allergology Unit, Bambino Gesù Children's Hospital IRCCS, Rome, Italy; s Center for Food Allergy and Asthma Research, Department of Preventive Medicine, Northwestern University Feinberg School of Medicine, Chicago, Illinois, United States; t Inflammation, Repair and Development Section, National Heart and Lung Institute, Imperial College London, London, United Kingdom; u Center for Environmental Medicine, The School of Medicine, The University of North Carolina at Chapel Hill, Chapel Hill, North Carolina, United States; v University of Colorado School of Medicine, Children’s Hospital Colorado, Aurora, Colorado, United States; w Pediatric Allergy, Immunology and Rheumatology Unit, Children's Hospital, Ain Shams University, Cairo, Egypt; x University Hospital Southampton, Southampton, United Kingdom; y Department of Paediatrics, The Medical University of Warsaw, Warsaw, Republic of Poland; z Children’s Allergy Service, Evelina Children’s Hospital, Guy’s and St. Thomas’ Hospital, London, United Kingdom; aa Department of Women and Children’s Health, School of Life Course Sciences, King’s College London, London, United Kingdom; ab Department of Pediatrics, Section of Allergy and Immunology, University of Louisville School of Medicine, Louisville, Kentucky, United States; ac Division of Immunology/Allergy Section, Department of Internal Medicine, University of Cincinnati College of Medicine, Bernstein Allergy Group, Bernstein Clinical Research Center, Cincinnati, Ohio, United States; ad Universidad Autónoma del Estado de Hidalgo, Pachuca, Hidalgo, México; ae Department of Allergy, Peking Union Medical College Hospital, Chinese Academy of Medical Sciences & Peking Union Medical College, Beijing Key Laboratory of Precision Medicine for Diagnosis and Treatment of Allergic Diseases, National Clinical Research Center for Dermatologic and Immunologic Diseases, Beijing, People's Republic of China; af Division of Allergy, Department of Pulmonology, Allergy and Thoracic Oncology University Hospital of Montpellier, Montpellier, France; ag Desbrest Institute of Epidemiology and Public Health, UMR UA11, University of Montpellier-INSERM, Montpellier, France; ah WHO Collaborating Centre on Scientific Classification Support, Montpellier, France; ai Institute for Immunological Research, University of Cartagena, Cartagena, Republic of Colombia; aj Medical Faculty at Akaki Tsereteli State University, National Institute of Allergy, Asthma & Clinical Immunology, KuTaisi, Tskaltubo, Georgia; ak EFA - European Federation of Allergy and Airways Diseases Patients' Associations, Brussels, Belgium; al Dartmouth Geisel School of Medicine, New Hampshire, United States; am Dartmouth Hitchcock Medical Center, Section of Allergy, Lebanon, New Hampshire, United States; an Section of Immunology, HIV and Allergy, Department of Medicine, Clinical Hospital University of Chile, Santiago, Chile; ao National Allergy Council, and Allergy & Anaphylaxis Australia, Sydney, Australia; ap Section of Inflammation, Repair and Development, National Heart and Lung Institute, Imperial College London, London, United Kingdom; aq Pediatric Allergy and Immunology Section, Department of Pediatrics, Hospital Británico de Buenos Aires, Buenos Aires, Argentina; ar Children's Hospital Colorado, University of Colorado School of Medicine, Section of Allergy and Clinical Immunology, Aurora, Colorado, United States; as Division of Paediatric Allergology, Department of Paediatrics, University of Cape Town, South Africa; at Universitat de Barcelona, Allergology and Clinical Immunology Department, Hospital Sant Joan de Déu, Barcelona, Spain; au Allergy Department, Second Pediatric Clinic, National and Kapodistrian University of Athens, Athens, Greece; av Division of Infection, Immunity and Respiratory Medicine, School of Biological Sciences, The University of Manchester, Manchester, United Kingdom; aw Allergy Unit, Hospital San Juan de Dios - Caja Costarricense de Seguro Social, San José, Republic of Costa Rica; ax Paediatric Allergy and Clinical Immunology, National Heart and Lung Institute, Imperial College London, London, United Kingdom; ay Heart and Lung Center, Department of Respiratory Medicine, Helsinki University Hospital and University of Helsinki, Helsinki, Finland; az Clinical Immunology and Allergy Division, University of Sao Paulo, São Paulo, Brazil; ba Department of Otolaryngology, Dar Al Shifa Hospital, Hawally, Kuwait; bb Department of Otolaryngology Head and Neck Surgery, Eye and Ear University Hospital, Beirut, Lebanon; bc Section of Allergy Immunology and Rheumatology, Department of Pediatrics, Centre Hospitalier Universitaire Sainte-Justine, Montréal, Quebec, Canada; bd Pediatric Allergy Unit, Department of Pediatrics, Gynecology and Obstetrics, University Hospitals of Geneva, Geneva, Switzerland; be Faculty of Health Sciences, Catholic University of Salta, Salta, Argentina; bf National Heart and Lung Institute, Imperial College London, London, United Kingdom; bg Northwestern University Feinberg School of Medicine, Ann & Robert H. Lurie Children's Hospital of Chicago, Chicago, USA; bh Sean N. Parker Center for Allergy and Asthma Research at Stanford University, Stanford University, Palo Alto, California, United States; bi Global Allergy and Airways Patient Platform, Vienna, Austria; bj Allergy and Asthma Network, Fairfax, Virginia, United States; bk Division of Population Medicine, School of Medicine, Cardiff University, Cardiff, United Kingdom; bl Allergy Section, Department of Internal Medicine, Hospital Vall d’Hebron, ARADyAL Research Network, Barcelona, Spain; bm Department of Internal Medicine, Seoul National University Bundang Hospital, Seoul National University College of Medicine, Seongnam, South Korea; bn Food Allergy Canada, North York, Ontario, Canada; bo Department of Woman, Child and General and Specialized Surgery, University of Campania 'Luigi Vanvitelli', Naples, Italy; bp Postgraduate Program in Allergy and Clinical Immunology, University of Naples ‘Federico II’, Naples, Italy; bq Division of Allergy and Clinical Immunology, Department of Medicine, ‘Santa Maria della Speranza’ Hospital, Battipaglia, Salerno, Italy; br Agency of Health ASL, Salerno, Italy

**Keywords:** Food hypersensitivity, Epidemiology, Economics, Anaphylaxis, Surveys and questionnaires

## Abstract

**Background:**

Data on severity of food allergy across nations are lacking. Building on the World Allergy Organization (WAO) DEFASE (Definition of Food Allergy Severity) score, we aim to explore its global applicability as a grading system for IgE-mediated food allergy (FA) severity.

**Methods:**

An international survey (WAO FASE Project) was conducted using an online questionnaire distributed to WAO members. The survey collected detailed data on diagnostic practices, therapeutic options, characteristics of FA patients, severity of reactions (including anaphylaxis), and eliciting doses of allergenic foods. In addition, FA management costs were examined (medical expenses, medication costs, and impact on quality of life and productivity).

**Results:**

We obtained information from 157 centers in 50 countries. FA management varied significantly across regions. Oral immunotherapy and omalizumab are widely used in Europe and North America. The use of advanced diagnostic tests (molecular diagnostics) vary widely between these regions. Thirty-five percent of patients with anaphylaxis exhibited severe symptoms (respiratory or cardiovascular compromise), with marked regional differences: more frequent in Western Asia (55.83%), Southern Africa (50%), and less frequent in South-Eastern Asia (12.5%) and Central America (21.72%). Approximately 1 in 4 patients reacted to less than half an age-appropriate portion of the allergenic food. Depending on the region, peanut, milk, egg, wheat, hazelnut, and peach allergies varied considerably. Economic resources and healthcare systems play an important role in determining access to diagnostic tests and therapeutic options, which have a direct impact on the severity and management of FA.

**Conclusions:**

With wide global disparities in access to diagnostic and therapeutic tools for food allergies, this condition entails a vast healthcare and economic commitment. The percentage of patients receiving a high severity diagnosis using DEFASE could be around 3%, similar to that of asthma patients diagnosed with severe refractory asthma.

## Introduction

With the development of new therapeutic options, the scientific community's attention towards food allergy has intensified.^1^ This health issue, which has been increasing in frequency for decades, has traditionally been managed with elimination diet.^2^ Recently, the possibility of protecting patients suffering from food allergy to oral immunotherapy has been accompanied by the possibility of protecting them from the consequences of inadvertent ingestion of food through omalizumab.^3^, ^4^, ^5^ As food allergy presents with a wide range of phenotypes and severity levels (ranging from oral allergy syndrome to fatal anaphylaxis), the new disease management modalities require careful consideration of candidates for individual therapies.^1^^,^^6^, ^7^, ^8^, ^9^, ^10^ Alongside the risks of side effects, the costs of the therapies and their effects on the natural history of the disease must be considered.

It is with cost-benefit ratios in mind that the World Allergy Organization (WAO) developed the Definition of Food Allergy Severity (DEFASE) project.^11^, ^12^, ^13^ A consensus of experts created a food allergy severity assessment grid similar to that available for asthma severity assessment.^14^^,^^15^ Essentially, it includes preliminary questions to exclude “difficult-to-treat” food allergy and, in those who do not display such issues, proposes to evaluate 5 severity criteria (Table 1).

**Table 1:** The DEFASE grid for food allergy severity

Difficult to manage issues: in this patient, proved difficult ….	Severity criteria (see also Figure S3)
… to accept her allergic condition	(A) Symptoms/signs with the most severe previous reaction
… to define triggering food allergen(s)	(B) Minimum therapy to treat the most severe previous reaction
… to avoid the triggering allergen(s)	(C) Individual minimal eliciting dose
… to read food labels	(D) Current impact on food allergy-related quality of life (FA-QoL)
… to be regularly followed up	(E) Current health-economic impact
… to get educated with her family	
… to carry self-injectable epinephrine	
… to understand and update a management plan	
… to be prepared to manage reactions	
… to properly treat a reaction	

The evaluation grid aims to include the emotional, social, and economical impacts of food allergy^16^ and its potential repercussions for health systems. The grid is being validated.^17^^,^^18^

Before its universal implementation, we wanted to assess the perception of clinicians regarding the items included in the food allergy severity evaluation grid. We also wanted to estimate the number of patients who would be diagnosed with severe food allergies after applying the DEFASE grid.

## Methods

An online questionnaire on Food Allergy Severity (FASE) was created and reviewed by members of the DEFASE panel, nominated by the WAO Food Allergy Committee. The protocol, approved by the WAO Executive Committee and Board of Directors, focused on the frequency and characteristics of food allergy in the respective countries (Supplementary appendix). It consisted of 42 questions under 4 major headings:

1 General questions regarding the respondent center (11 questions),
2 Specific questions about the how milk/egg/peanut diagnosis and treatment are delivered and the characteristics of food-allergic patients at the respondent center (11 questions),
3 A specific question about the difficult-to-manage food allergy issues experienced by patients at the respondent center, and
4 Information on food allergic patients in the country to which the center belongs (19 questions).

The questionnaire included a definition of anaphylaxis (“… a serious systemic hypersensitivity reaction that is usually rapid in onset and may cause death”) and its severity (“… severe anaphylaxis is characterized by potentially life-threatening compromise in the airway, breathing and/or the circulation, and may occur without typical skin features or circulatory shock being present, requiring prompt identification and treatment”).^19^ Since there are multiple definitions of the severity of the manifestation of anaphylactic episodes, we instructed the responding centers to use the table generated in the DEFASE systematic review (Figure S1)^12^ to define the severity of anaphylactic reactions presented in the past by patients with food allergy. To illustrate the concept of “very small” eliciting doses, our questionnaire also included a table of the age-appropriate portion sizes for different food allergens.^20^

The questionnaire was developed as a web-based survey using SurveyMonkey®, in English, and was circulated to the representatives of the 95 constituent national societies of WAO in November 2021. It was also administered to the WAO Centers of Excellence involved in food allergy. This sampling frame consisted of all members of the 95 national societies. It included not only allergists who deal with food allergy, but also all those who deal with respiratory, drug, and hymenoptera allergy. As the questionnaire contained an express invitation to respond only for those who deal with food allergy, our target population was just a fraction of the 37,000 questionnaire recipients.

A reminder was launched after 21 days to re-engage potential respondents.

## Results

### Participating centers

**Fig. 1:**
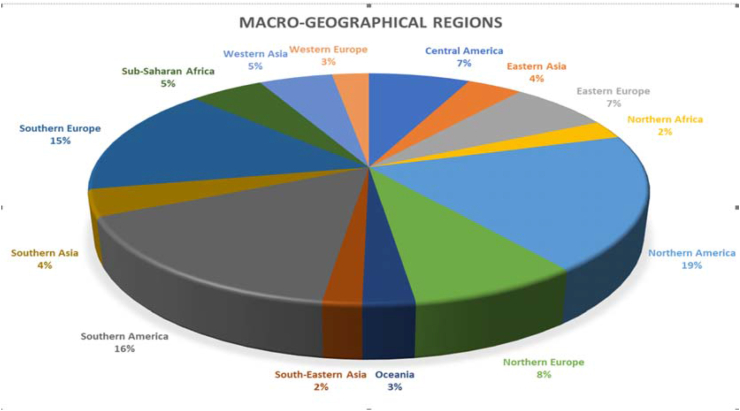
Geographical distribution of the responders by UN macro-geographical regions.

We received 157 responses from 50 countries [Fig. 1; supplementary table S1]. The majority of respondents (57) were operating in the context of a universal government-funded (ie, “single-payer”) health system, 29 in a non-universal insurance system only, 20 in a universal public-private insurance system, and 5 in a universal private health insurance system. All the others were operating in mixed systems, mainly in non-universal insurance system + universal government-funded health system (10 respondents). Specifically, concerning the activities of the responding centers, 59 carried out exclusively public practice, 45 exclusively private practice, and 53 operated mixed public/private practice (Table 2). The percentage of centers operating public practice was maximum in Northern Europe (84.6%) and minimum in South-Eastern Asia (none).

**Table 2:** Type of assistance provided by the respondents’ centers

Word region	Type of assistance			
	Public practice	Private practice	Both	Total
North America	9	5	16	30
Central America	2	5	4	11
South America	10	10	5	25
Northern Africa	1	1	2	4
Southern Africa	1	4	3	8
Northern Europe	11	1	1	13
Eastern Europe	4	3	4	11
Southern Europe	9	4	11	24
Western Europe	2	1	1	4
Oceania	3	1	0	4
Western Asia	3	2	3	8
Eastern Asia	3	0	3	6
Southern Asia	1	5	0	6
South-Eastern Asia	0	3	0	3
Overall	59	45	53	157

Diagnosis through skin testing is universally widespread with some deficiencies in sub-Saharan Africa, Southeastern, Eastern, and South Asia (Table 3). Almost all centers are able to offer the determination of total and specific IgE. In the regions where the skin prick test is not universally available, the diagnosis of sensitization through determination of specific IgE is more frequently reported.

**Table 3:** Diagnostic and therapeutic tools offered by the respondents’ centers (% of respondents for each region)

Offered tool	North America	Central America	South America	Northern Europe	Eastern Europe	Southern Europe	Western Europe	Western Asia	Eastern Asia	Southern Asia	South-Eastern Asia	Oceania	Northern Africa	Southern Africa
Skin prick tests	100	100	84	100	90.9	95.8	100	100	66.7	66.7	33.3	100	100	50
Total IgE determination	96.7	54.5	84	92.3	90.9	91.7	100	100	100	50	66.7	83.3	75	87.5
Specific IgE determination	96.7	36.4	68	92.3	90.9	91.7	100	100	100	50	66.7	100	50	62.5
Molecular IgE determination	50	27.3	32	76.9	81.8	83.3	75	50	50	16.7	33.3	83.3	0	12.5
Diagnostic OFC	93.3	45.5	56	84.6	72.7	70.8	50	75	66.7	0	0	100	50	37.5
OIT	63.3	9.1	24	46.2	27.3	45.8	75	25	16.7	0	0	16.6	25	25
Omalizumab	80	36.4	36	76.9	18.2	75	100	50	66.7	16.7	0	83.3	75	0
Dupilumab	76.7	18.2	24	38.5	0	62.5	75	25	50	0	0	66.6	0	0

The use of molecular diagnostics seems to be for the moment confined to specific regions, in particular in Europe and Oceania, less frequent in North America, Western Asia, and the Eastern Asia, while it is rarely available in Africa, Central America, and South America. As these centers were selected for their competence in food allergies, it is not surprising that many centers are able to offer diagnostic Oral Food Challenges (OFCs). Oceania stands out, with a universal availability of OFCs, followed by North America, Northern Europe, and Eastern Europe. The respondent centers in Western Europe do not exceed 50% for this diagnostic service, also surpassed by South America with 56%.

Oral immunotherapy is a widespread practice mainly in Western Europe, followed by North America, Southern Europe, and Northern Europe. In all other contexts the availability of this practice does not reach 50%. Many centers in Western Europe, North America, Eastern Asia, and Oceania reported using omalizumab for the treatment of food allergy. Many centers, mainly in the same regions, also have dupilumab available, and report that they have thought about its use also in the context of food allergy.

### Patients, food allergy, and its severity at the participating centers

Among the respondent centers the largest were those in Western Europe with an average of 4000 patients per year, followed by North Africans (1778), North Americans (1400), Northern Europeans (1366), Southern Europeans (833), Middle Eastern (600), Central Americans (538), Eastern Europeans (505), South East Asians (351), South Americans (322), Australians and New Zealanders (250), and South Asians (217). Southern Africa reports a mean 180 patients per year, while we do not have data for East-Asian centers. The overall mean of 853.86 (±1441.84) per year indicates that these centers see a considerable number of food allergy patients.

**Fig. 2:**
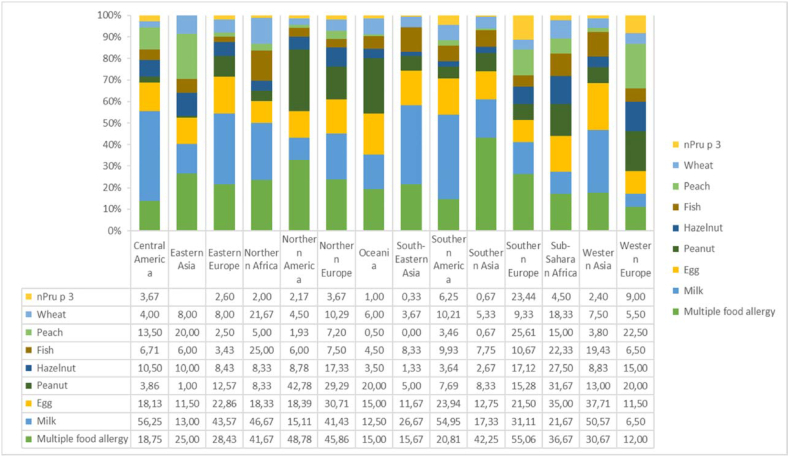
Frequency of allergy to specific foods in different regions of the world as reported by the responding centers.

Our survey captures an interesting difference between the types of foods reportedly responsible for food allergies in different parts of the world. As already suspected, peanut was the common food allergen in North America. Peanut allergy was also frequent in Northern Europe, Oceania, and Western Europe (Fig. 2). Peanut allergy is reported in only 5% of patients from South-East Asia, 3% of those from Central America and 1% of patients from the Eastern Asia. The remarkable difference in frequency of cow's milk protein allergy may reflect the different composition of the populations belonging to our polled centers, but also the different demographic composition of the different populations of the world. In countries with lower average age, milk allergy was more widespread: 56% in Central America, 54% in Western Asia, 50% in South America, 46% in North Africa, and 43% in Eastern Europe. Milk allergy is reported less common in the population of North America (15%), Eastern Asia (13%), Oceania (12.5%), and Western Europe (6.5%). At least in this latter context, characterized by systems offering pediatric assistance in an universalistic frame, it is very likely that milk-allergic children are managed by primary care pediatricians where there is a specialist in the area, and therefore do not come under the observation of allergy centers except in the most serious cases.^21^ Egg allergy is also not uniformly distributed, with 37% of patients in Western Asia and 35% of patients in Southern Africa reported allergic to this food, dropping to 31% in Northern Europe, 23% in South America, 22% in Eastern Europe, 21% in Southern Europe, down to 18% of Central America, North America, and North Africa. The lowest egg allergy frequency (11.5%) is reported in Western Europe and Eastern Asia. There are no regions where this allergy did not reportedly affect at least 10% of the responding center's food allergy patients. Wheat allergy was more prevalent among patients at responding centers in Northern and Southern Africa (21.6% and 18.3%, respectively) compared to responding centers in European, Asian, and American regions. Of note, another unequally distributed allergy is hazelnut, which seems more prevalent among patients presenting to Southern African and European centers, while it is less frequently reported among patients seen by centers in East Asia and Central America and is practically unreported in South Asia and South-East Asia as well as in South America. Peach allergy is prevalent in Southern Europe, where it reaches 25.6%, followed by Western Europe and Eastern Asia. It is interesting to observe how Pru p 3 allergy, linked to peach allergy,^22^ is almost universally reported in Southern Europe and much more rarely in other regions, including those where peach allergy is reported. This finding possibly reflects the difficult access to molecular diagnostics in some regions of the world.

Another aspect of great interest is the different frequency of multiple food allergies, reported at almost 50% in North America, Northern Europe, and only 12% in Western Europe and 15% in Oceania.

The marked diversity of manifestations observed in the different centers may be at least partially linked to the different frequency of sensitization to individual foods, as can be seen in Table 4. In general, the most frequent of the manifestations reported is food-induced urticaria, but the second in order of frequency is food-induced anaphylaxis. Our polled centers report about 25% of patients with this type of manifestation, indicating that at least in some regions the cases of more severe food allergies are concentrated in these centers.

**Table 4:** Percentage of symptoms experienced by food-allergic patients across regions in respondent centers (given the possibility of multiple symptoms, the total can exceed 100)

Clinical presentation(s)	North America	Central America	South America	Northern Europe	Eastern Europe	Southern Europe	Western Europe	Western Asia	Eastern Asia	Southern Asia	South-Eastern Asia	Oceania	Northern Africa	South Africa	Overall
Food-allergic G.I. symptoms	24.4	46.7	38.7	13.3	30.3	25.4	6.5	30	15	0	0	20	25	30	**28.36**
Food-induced oral allergy syndrome	22.5	36.3	17.2	25.4	25.4	39.7	37.5	29.7	25	8	1.7	10.5	33.3	6.7	**25.24**
Food-allergic rhinitis	4.7	5.4	11.4	10.8	6.8	14	3.5	15.4	10	25	8.3	1	16.7	12.5	**10.50**
Food-allergic asthma	5.7	6.6	8.6	10.8	6.5	12.8	3.5	16.9	10	25	0	3.5	13.3	17.5	**9.62**
Food-induced anaphylaxis	48.9	4.6	18.7	32.5	12.1	22.7	11.5	16.9	5.5	7	27	42.5	11.3	33.3	**24.05**
Food-allergic atopic dermatitis	11.3	23.7	25.2	30	40.7	18.6	6	42.6	31.5	35	23.3	13.5	41.7	27.5	**24.72**
Food-allergic immediate urticaria	49.2	17.9	58.7	36.4	19.4	47.5	17.5	41.1	50	46.5	36.7	41.5	34	73.3	**43.77**
Other	20.2	3	7.20	0	20	15	25	0	0	58	0	30	0	0	**17.76**

Less severe food allergies were also cared for in our polled centers. Oral food allergy syndrome was reported in 25.2% of cases, mainly in South-Western Europe and in Central America. Gastrointestinal symptoms were reported in 28.3% of cases. Atopic dermatitis was reported in 24.7% of cases, respiratory manifestations were rarer. The cases of anaphylaxis were more frequent in North America (48.9%), Northern Europe (32.5%), and Oceania (42.5%), indicating that in these countries the mix of food allergy patients in the respondent centers is selected upwards. In other regions, relatively fewer food allergy patients presented with anaphylactic reactions: in Central America 4.6%, Eastern Asia 5.5%, South Asia 7%, and North Africa 11%. Surprisingly, among these regions we also find Western (11.5%) and Eastern Europe (12.1% of patients reported with anaphylaxis).

As the characteristics of the symptoms with the most severe previous reaction are part of the DEFASE grid, in our questionnaire we tried to evaluate the severity of the anaphylactic manifestations presented by patients from our polled centers. What emerges is that 11.7% had anaphylaxis with respiratory or circulatory failure, 26.2% manifested laryngeal, asthmatic or cardiovascular symptoms, while 64.7% experienced true anaphylaxis (involving many organs), but with the presence of only skin lesions and mild to moderate gastrointestinal symptoms or rhino-conjunctivitis (Table S2). Therefore, with reference to the overview of anaphylaxis symptom-severity scores ordered by organ (Figure S1), 64.7% of the reported patients can be placed on the left side (green-yellow symptoms), while 35.3% presented manifestations of severe anaphylaxis (red symptoms) and fell into the right side. The "red symptoms” anaphylaxis are more frequent in Western Asia (55.83% of the reported anaphylactic food allergies), Southern Africa (50%), Western Europe (43%), Eastern Europe (41.2%), and North Africa (40%). The percentages of patients with severe manifestations of anaphylaxis reach 39.38% and 39.18% in North America and South America, respectively. They are 38.64% in Southern Europe, 35% in Oceania, and 32.9% in Northern Europe. The percentages of reported severe anaphylaxis are lower in South-Eastern Asia (12.5%), Central America (21.72%), Southern Asia and Eastern Asia (30% and 30.5%, respectively) (Table S2).

Among patients reported to have anaphylaxis, the percentage of those requiring adrenaline in the most severe of the past anaphylactic reactions was reported between 3% in South Asia and 50% in Southern Africa. Epinephrine was also used frequently in East Asia (47%) and Western Asia (38.5%), Southern Europe (35%), North America (34.5%) and Western Europe (27%). Patients with anaphylaxis got adrenaline quite rarely in South Asia and North Africa (Table S3).

The mean percentage of severe anaphylactic reactions for which repeated doses of adrenaline were used is 4.6%, and this was more frequently reported in West Asia and Eastern Europe. The reported proportion of patients requiring multiple doses of adrenaline among those treated with adrenaline varied widely across regions. This was most frequently reported in Eastern Europe (62%). This practice is also frequent in Northern Europe (36%). The use of multiple doses of adrenaline occurs in 29.8% of the most severe past anaphylactic reactions in Western Asia, in 20.7% in Western Europe, 15% in North America. Table 5 shows the correlations between percentages of patients reported with severe anaphylactic reactions and use of adrenaline, which are not significant.

**Table 5:** Percentage food-allergic patients experiencing severe anaphylactic reactions, of use of adrenaline and of use of multiple adrenaline in the most severe previous anaphylactic reaction across regions in respondent centers. R (a) vs. (b), 0.596; R (a) vs.(c), - 0.110

Word region	% severe anaphylaxis	% adrenaline users in the most severe anaphylactic reaction	% of 3 or more adrenaline users in the most severe anaphylactic reaction
	(a)	(b)	(c)
Western Asia	55.83	50.05	11.50
South Africa	50	56.25	6.25
Western Europe	43	32.50	5.50
Eastern Europe	41.2	26.67	10.67
North Africa	40	13.5	0.50
North America	39.38	39.67	5.17
South America	39.18	24.97	2.50
Southern Europe	38.64	21.61	2.50
Oceania	35	27.50	3.00
Northern Europe	32.9	13.67	3.67
Eastern Asia	30.5	47.50	7.50
Southern Asia	30	3.33	0
Central America	21.72	24.47	5.33
South-Eastern Asia	12.5	12.50	0

Since the dose eliciting a reaction to foods is a severity criterion agreed within the DEFASE consensus, we wanted to verify what proportion of patients reacted to small and very small doses according to the responses of the polled population. The amount of anaphylactic food reactions that are caused by very small doses is 16.5%, corresponding to approximately 1 reaction in 6 (Table S4). One reaction in 4 is caused by doses less than half the average portion. Overall, for more than 50% of the anaphylactic patients reported in our sample the food-triggering dose in the most severe food-allergic reaction was due to small or very small quantities of food. This data, in addition to calculating the number of patients with severe food allergies, are relevant to figure out the proportion of anaphylactic patients for whom the avoidance of food traces should be recommended. Given the current legislative uncertainty, it appears clinically sound to suggest this behavior to approximately half of children with food allergies who develop anaphylaxis.

**Fig. 3:**
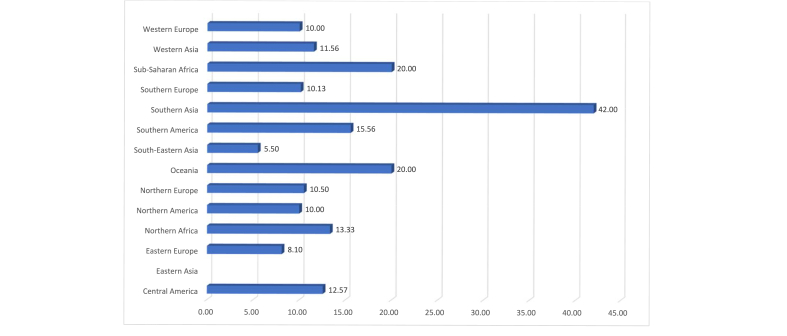
Percentage of patients reported with more than 1 severe reaction per in the last 12 months, by region

There are no apparent correlations between the frequencies with which anaphylaxis is reported in the different regions and the frequency with which it has occurred repeatedly. The recurrence of episodes of severe anaphylaxis in the same patient in the last 12 months varies between 5.5% and 20%, with a remarkable 42% in South Asia (Fig. 3). This finding may be linked to a difficulty in managing nutrition education in some regions of the world including Southern Africa, North Africa, Middle East, and Central America.

However, the recurrence of severe events remains present in all regions of the world, signaling the need to act in preventive terms both at an individual and community level to reduce the risk factors of anaphylactic reactions. An exception is Japan, where no repetition of severe anaphylaxis events was reported in the same patients in the last 12 months.

In our survey, the pooled centers were asked to estimate the impact of food allergies on the quality of life including the emotional impact, dietary and social limitations. For 1 patient in 5 only it was reported that their food allergy minimally impacts their quality of life. For all the others the impact was described as moderate or significant (Table S5). Everywhere a significant impact was reported in at least 15% of patients, but in Eastern Europe, Central and South America, South Asia, Western Asia, Southern Africa, and Central America the quality of life was described severely affected for more than 40% of patients. Also, in this field the pleasant Japanese exception stands out, where only 1% of food allergy patients were reported to suffer a significant influence on their quality of life.

### Difficult to manage food allergy issues at the participating centers

For the purposes of FASE, the questions were grouped in 5 categories (Table 6). The following percentages of anaphylactic patients were reported with difficult to manage issues.

I) difficulty to avoid triggering allergen(s), 20.5%,
II) difficulty to define triggering food allergen(s), 21.6%,
III) difficulty to educate patients and family, 17.8%,
IV) difficulty to be prepared to manage reactions, 20.5%, and
V) difficulty to properly treat a reaction, 23.8%.

**Table 6:** Percentage of food-allergic patients reported with difficult to manage issues across regions in respondent centers

Failure to (%): …	North America	Central America	South America	Northern Europe	Eastern Europe	Southern Europe	Western Europe	Western Asia	Eastern Asia	Southern Asia	South-Eastern Asia	Oceania	Northern Africa	Sub-Saharan Africa	Overall
… avoid the triggering allergen(s)	15.65	31.25	20.22	12.50	29.43	20.06	12.50	27.14	9.00	20.00	28.33	18.67	18.33	13.33	**20.51**
… define triggering food allergen(s)	13.94	24.38	24.40	15.00	20.86	26.22	17.50	19.67	10.00	21.25	30.00	13.67	40.00	35.00	**21.66**
… educate patient and family	12.78	27.00	20.50	20.00	11.57	18.47	10.00	26.33	9.00	20.00	23.33	5.00	5.00	23.33	**17.85**
… be prepared to manage reactions	15.59	30.63	30.44	7.20	18.14	17.82	10.00	24.43	2.50	25.00	26.67	7.67	14.00	23.33	**20.51**
… properly treat a reaction	24.47	30.63	34.56	11.00	12.29	21.18	17.50	23.29	1.50	26.00	6.67	26.67	18.33	28.33	**23.37**

Issue (I) was less reported in North America, Japan, Southern Africa, Western Europe and Northern Europe (9%–15.6%, compared to 18.2%–31.2% in Northern Africa, Latin America, Southern Asia, and Western Asia). Issue (II) was more reported in Africa (40%) and least reported in Japan (10%). Issue (III) exceeded 20% in South-Eastern Asia, Western Asia, Southern Africa, and Latin America. Issues (IV) and (V) were less frequent in Japan, followed by Europe. Difficulties remain in other regions, including North America.

#### Information on food allergic patients in the respective countries

A batch of questions was aimed at detecting the respondents' perception of the secular trend of food allergy in their territory. Specifically, it was asked if, in the last 10 years, the frequency of food allergy has increased, decreased, or remained stable. To that question, 55.4% of the centers responded, with 90% of reporting an increase in the prevalence of the condition. These data were mostly estimated based on personal experience of changes in clinical service burden or health care service activity. In 18.4% cases, published epidemiological evidence was available for the specific country. The majority of these respondent centers (71.2%) also reported an increase of food allergy severity among the national population, although published evidence was available in 13.8% of cases only.

The following set of questions was aimed at understanding the frequency of food allergies at different ages. The response rate for these questions was 63.7%.

**Fig. 4:**
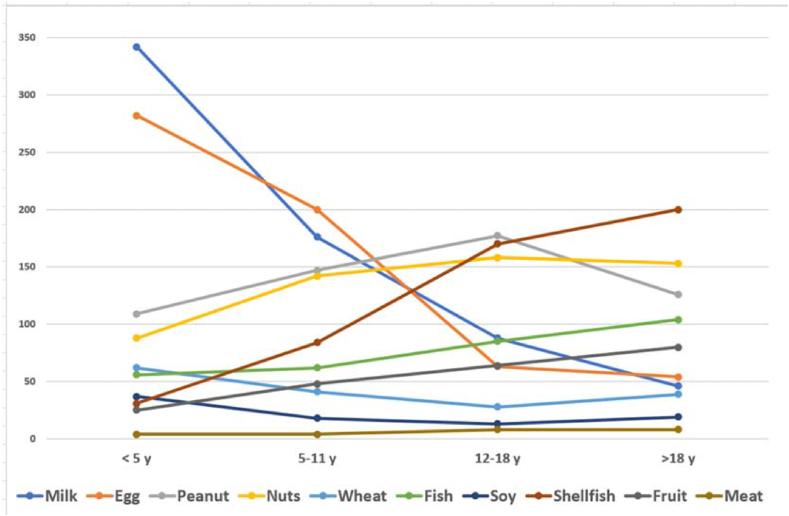
Food allergy elicitors by age (reported by frequency in FASE survey).

The prevalent allergens in children under 5 years of age were found to be milk and egg, considered first or second allergen by 88%, and 84% of the responding centers, respectively (Fig. 4, table S6). Other allergens reported to be of maximum prevalence in some countries were peanuts (15%, especially in English speaking countries), wheat (3%), fish (3%), soy and fresh fruit (2% each). Second-line allergens in this age group (ranked third of fourth by frequency) are nuts (45%) and wheat (37% reports). Where it is not among the top 2 allergens, peanut is permanently in third or fourth place. Fish is reported to be an important allergen, especially in South-East Asia and South Asia with 32% citations as the third or fourth allergen, while seafood is reported in third or fourth place in 20% of cases.

Things change between the ages of 5 and 11. At this age the prevalence of egg allergy as first or second allergen (51%) exceeds that of milk (42%), then peanut and tree nuts pair with 33%, shellfish at 10%, and fish at 8% (table S7). Less frequent allergens are wheat (first or second in 3% of cases only, but in third or fourth position in 13% of cases), fruit (third or fourth position in 19% of cases), and peach (4%, all from the Mediterranean area).

In adolescents between 12 and 18 years old, the most common allergen globally is peanut with 50.8% of responses, followed by tree nuts with 40% of responses and shellfish with 36.9% of responses (Table S8). In this age group, fresh fruit and fresh vegetables are also among the most frequent allergens with 13.8% responses, while the percentage of centers reporting milk (15.4), egg (9.2%), and wheat (4.6%) as relevant allergens in this age phase is significantly reduced. In third - fourth place in this age group we found fish (29.2% of the answers), and shellfish (30.7%). It is interesting to note that some centers report a significant incidence of reactions to seed oils, especially considering the non-uniform worldwide distribution of legislation on allergies to these foods.

In the evaluation reported by our responding centers, the highest prevalence of food allergy in adults is for shellfish, reported to be the first or second allergen in 33 cases, followed by nuts reported in 23 cases, peanut (19), fish (16), and fresh fruit (11 cases). In this age group, the percentage of allergy among the first 2 food allergens was lower for egg (7 cases) and milk (6 cases) (Table S9). Where tree nuts and shellfish were not the 2 major allergens, they were cited as the third, fourth or fifth allergen practically everywhere. Other allergens reported less frequently were fish, wheat, vegetables, and legumes. Only 1 center reported mustard as a relevant allergen, and no one sesame; however, at the time of the survey, sesame was not listed separately from other nuts in the Codex General Standard for the Labelling of Prepackaged Foods (GSLPF).^23^^,^^24^

It should be noted that the percentages estimated by the centers refer to personal experiences in the majority of cases. Only in a quota varying between 4.82% and 10.85%, depending on the age groups, did the data refer to published studies on the epidemiology of food allergy (Tables S6 – S9).

The report of the different health costs of services and therapies for food allergy (Table 7) were provided in local currency and converted into United States dollars at the exchange rate of June 30, 2024. This data require some comment already in the phase of presentation of the results. The cost of an administration of adrenaline is extremely variable between one region and another. Only 67.8% of respondents declared that self-injectable adrenaline is available in their country. Where adrenaline auto injectors are present, they are provided without cost to the patient by the national health system in 77.2% of cases. Standardized national anaphylaxis action plans area available in 44.1% of centers (Table S10).

**Table 7:** Unit cost of different healthcare services resulting from food allergy across regions in respondent centers.

Word region	Adrenaline^a^	ICU^b^	ED visit	Call^c^	ED^d^	Dietician^e^	OFCͰ^f^	SPT ×7 g	Mol ×12 h	RAST ×7 i	Paed j	GP k	Psych l	Allergist m	Total
North America	212.54	4240.00	1254.80	1537.00	1756.44	96.34	2057.22	96.85	329.41	172.65	124.20	108.58	134.93	183.58	12,304.55
Central America	1.92	1283.41	199.53	76.42	605.37	23.93	210.56	75.10	140.28	82.92	25.47	19.27	24.84	35,96	2804.99
South America	182.50	416.01	60.19	57.21	191.52	29.85	95.60	34.79	168.34	49.56	37.92	34.10	31,63	47,32	4115,48
Northern Europe	107.39	1529.88	53.16	0.00	41.45	25.40	211.28	16.00	0.00	60.00	7.05	31.40	11.75	51.28	12,932.88
Eastern Europe	40.80	34.00	55.65	42.00	139.65	29.28	50.46	28.24	52.95	30.28	20.90	16.11	41.80	41.94	5266.83
Southern Europe	39.71	924.62	146.43	84.23	315.36	52.00	187.08	40.61	133.07	53.23	32.32	23.21	47.00	76.68	2155.55
Western Europe	95.00	250.00	62.50	100.00	62.50	47.50	110.00	17,50	77.50	42.50	15.00	12.50	55.00	15.00	882.50
Western Asia	34.71	349.00	32.31	92.06	113.63	36.88	1971.87	212,24	214.93	94.33	44.66	27.89	37.24	50.65	3312.39
Eastern Asia	148.50	1130.47	21.45	265.23	433.78	11.55	148.50	60,78	165.00	0.00	4.95	4.95	48.86	17.82	2461.84
Southern Asia	60.00	380.00	42.80	50.00	132.00	10.80	24.00	40,00	156.00	54.80	13.20	9.20	11.20	26.00	1010.00
South-Eastern Asia	30.00	245.00	150.50	84.00	727.50	19.00	68.00	49.00	177.50	54.50	24.50	16.80	38.00	38.00	1722.30
Oceania	73.17	781.67	223.33	402.00	223.33	44.67	134.00	43.55	122.83	21.22	92.68	14.52	123.53	92.68	2393.18
North Africa	18.20	126.53	13.87	18.20	52.00	13.00	18.20	11.27	277.33	26.87	6.93	8.67	22.53	14.73	628.33
Southern Africa	29.37	520.00	72.67	80.00	269.67	10.40	87.00	15.95	145.00	43.48	31.70	15.97	17.17	25.90	3165.55
Mean	**95.61**	**1130.47**	**244.27**	**265.23**	**433.78**	**39.41**	**613.84**	**60.78**	**159.96**	**68.62**	**41.03**	**31.78**	**48.86**	**66.17**	

a Adrenaline (/epinephrine) administration; where available, cost of an adrenaline (epinephrine) auto-injector.

b One day spent in ICU because of food allergy.

c Cost of an emergency ambulance call.

d Cost of an Emergency Department visit, # Cost of an Emergency Department admission up to 6 h.

e Cost of a dietician consultation.

f Cost of an Oral Food diagnostic Challenge.

g Cost of a diagnostic skin prick test (7 extracts).

h Cost of a molecular diagnostic test (12 allergenic molecules).

i Cost of a serum test panel (7 food allergens).

j Cost of a community visit to general paediatrician.

k Cost of a community visit to general practitioner.

l Cost of a community visit to general practitioner.

m Cost of a community visit to an allergist.

Where self-injectable adrenaline is not available, the cost of administering a dose of adrenaline using a traditional syringe was reported to be between $1.92 and $18.20. The reported costs of self-injectable adrenalines varied from $30 in Southern Africa, Western Asia, South Europe (Greece, Portugal, Italy, and Spain) up to a cost of $212 in North America. Thus, a wide disparity in costs of auto-injectors across different countries is in place. The cost of an ambulance call is equally different, varying between $18 reported in North Africa and $1500 reported in North America. An emergency room visit has a minimum cost of $13.8 in North Africa, up to $1254.8 in North America. The variations in cost of these services are partly linked to the type of healthcare system in which the food allergic patient is assisted. Being held in the emergency room has a cost for the individual or the community varying between $52 in North Africa and $1756.44 in North America. When the reaction is as serious as to involve hospitalization in intensive care, the costs of a day of hospitalization range from $126.5 in North Africa to $4240 in North America. European costs are placed in an average between these variables, with a notable low price in Eastern Europe where we had respondents from Poland, Romania, Moldavia, Bulgaria, and Russia. We assume that the costs to which our responders refer are in this case simply the costs of participation borne by the patient in universalistic health systems.

The basic food allergy diagnostic services present fewer variations at a global level. The cost of skin tests for allergy diagnosis varies between $11.27 in North Africa and $96.85 in North America for 7 food allergens. The cost of specific IgE dosage for 7 foods varies between 21.20 dollars in Oceania and 172.65 in North America, and that of molecular diagnostic tests for a panel of 7 allergens is reported varying between $77.5 in Eastern Europe and $329.41 in North America.

The cost of specialist healthcare services was recorded for dietician ($10.4 in Southern Africa - $96.3 in North America), the pediatrician ($4.90 in Eastern Europe - $124.20 in North America), and the general practitioner whose cost varies between $8.60 in North Africa and $108.60 in North America. If the patient needs a psychological evaluation, she can expect to spend from $11.7 dollars in Northern Europe up to $135 in North America. An allergy visit can be obtained for patients at a cost ranging from $25.9 in Southern Africa up to $183.5 in North America. Finally, the cost of the oral provocation test was reported to be trivial in North Africa with an expense of $18.2 (but we know that this region lacks facilities for OFC), and high in North America with a cost of $2057.2.

## Discussion

The survey collected data from 14 of the 19 regions belonging to the United Nations scheme.^25^ The regions not represented were Central Asia (Kazakhstan, Kyrgyzstan, Tajikistan, Turkmenistan, and Uzbekistan), Polynesia, Melanesia, Micronesia, and Antarctica.

In the represented regions, allergy appears to be a bridge specialty between public and private practice. Even in universalistic systems, such as the National Health System (NHS) of the United Kingdom and the Italian Servizio Sanitario Nazionale (SSN), it is practiced in both contexts. This indicates that, despite the social alarm that food allergy is generating, the resources allocated by national health systems do not generally seem to cover the patient needs, except in some (predominantly Northern European) countries. The varied situation described a few years ago^26^, ^27^, ^28^, ^29^, ^30^, ^31^ persists.

The size of the centers seems rather large especially in European and North American areas. Facilities in South America, Africa, South-East Asia, and the Middle East report a lower number of patients per year. However, all centers manage a large number of patients, indicating that in different countries the management of food allergies tends to be concentrated in reference centers.

The responding centers offer diagnostic services adapted to their respective realities, dietary suggestions to avoid food allergens and therapeutic solutions in case of accidental reactions. Beyond these facilities, however, the high percentage of the centers reporting use of biological therapies for food allergy indicates the need, or at least the universal aspiration, to take advantage of food allergy therapies that are not limited to simple avoidance of the allergen. We believe that the research fervor in this area is generating expectations among the allergy community: already in 2023, before the approval of food allergy indication in the United States, our polled population was informed about the trials and observational studies indicating high efficacy and tolerability of the use of omalizumab for the treatment of food allergy.^32^, ^33^, ^34^, ^35^, ^36^, ^37^

At the time the questionnaire was conceived, studies were underway to verify the potential usefulness of dupilumab in food allergy.^38^^,^^39^ The substantially negative conclusion of these studies makes this part of our answers anachronistic.^40^^,^^41^ We believe that if the questionnaire were administered again today, no one would think of using this drug for the management of food allergies.

Some of the survey results allow to draw inferences about the severity classification that could be applied to the populations referred using the DEFASE grid.

### Difficult-to treat food allergy

DEFASE is the first consensus in which food-allergic patients with difficult-to-treat problems have been identified as a specific category. This category, already well known for patients with asthma,^42^ includes all the critical issues of diagnosis, education and preparation of the patient and family, the correction of which could substantially improve the patient's condition and reduce the subsequent severity classification. In this phase of the questionnaire, the clinician was solicited to verify with the parents or the patient himself a series of pre-requisites before filling the severity score. Is diagnosis complete? Does the patient know every possible culprit food allergen? Is the patient, parent and/or her family fit for food allergy? Is she skilled in managing her allergic condition in terms of regular follow-up, goal setting and problem-solving? Is she prepared to manage reactions? Is the management plan updated? Is the patient willing and able to avoid the triggering food allergen(s)? Is she able to interpret labels? Is the patient willing and able to carry self-injectable epinephrine? Is she prepared to properly treat a reaction? Only if all the answers are “yes”, the food allergy severity is estimated. If not, the suggestion is to correct the issue(s) before scoring severity.

The results of the questionnaire showed a situation that was not unexpected. In none of the responding centers are managing difficulty issues absent. They vary between 5% of educational difficulties reported in Oceania and 40% difficulty in identifying the allergen in North Africa. Even where a high standard of care is provided, patient and family education continue to be problematic in up to 27% of cases, as reported in Central America.

The managers of the responding centers consider that, despite the education provided, not all patients are prepared to manage reactions. This includes prompt recognition of symptoms, management of emotional stress, implementation of action plans, and auto-injector training.^43^ The best situation appears to be reported in Japan, while clinicians from Central and South America, as well as those from South Asia and even Southern Africa consider a significant proportion of their patients unprepared to manage and treat reactions in practical life.

The definition of trigger allergens appears to be better where there are stringent legislation on food allergen labelling as in Japan, Europe and North America.^44^ Where such legislation is not present, as in North Africa, it may become difficult for patients to recognize the respective sources of danger. Even if they are recognized, there are regions where it is difficult to avoid the trigger allergens.

The most frequent of the difficult to manage issues is the ability to appropriately treat a reaction. There are levels of misunderstanding in the management of situations of food allergic people that depend on their cultural level, risk perception, on the multiplicity of their allergies, or environmental factors, which are certainly incompressible,^45^ but our snapshot of the situation identifies many possible areas of improvement in the management of food allergy across the regions of the world.

### Symptoms/signs with the most severe previous reaction

The number of patients reported with respiratory and circulatory failure during the most severe previous anaphylactic episode was 11.7%. As the reported incidence of anaphylaxis was 24.05%, the fraction of patients who receive the highest score in Domain A (figure S3) was 2.8%. The data reported in Table 4 suggests that the severity of severe anaphylactic manifestations in different regions may be different. In fact, the lowest number of food allergy patients with a history of anaphylaxis was reported in Central America followed by South Asia, East Asia, and North Africa. On the contrary, anaphylaxis causing circulatory or respiratory failure was reportedly more frequent in North America (14% of patients with food allergies), Western Asia (5.3%), Northern Europe (4.4%), Eastern Europe (3.5%), Northern Africa (3.4%), and Oceania (3.1%). These are therefore the regions in which one can expect to see the greatest number of patients with a 3-point score in Domain A. Our data suggest a possible lower frequency of such subset in South-Africa (2.3%), Southern America (2.6%), Southern Asia and Southern Europe (1.7%), Western Europe (1.5%), Eastern (1.1%) and South-eastern (0.5%) Asia, and in Central America (0.7%).

### Minimum therapy to treat the most severe previous reaction

The use of adrenaline in the most severe of the past anaphylactic reactions was reported most frequently in Western Asia, Southern Africa, North America, and Eastern Asia, less frequently in other regions; quite rarely in South Asia, Southern America, and North Africa (table S2). It has been described that the regions in which adrenaline as auto injectors is less available are North Africa (10% of cases as an imported product only), South America (missing in 72% of cases), and Asia-Pacific in which it is presently missing in 41% of cases.^46^ Since in these regions the auto-injectors are little prescribed, it is perhaps no coincidence that these are the regions in which the use of adrenaline is less reported even in the most severe anaphylactic reactions. The auto injectable adrenaline is available instantly and can be self-administered by the patient himself, while the adrenaline for preparation is generally administered during emergency room visits. The data reported underline the importance of making adrenaline available worldwide as a life-saving device, without which the risks for people with food allergies must be considered increased.

In our opinion, even in regions where the reported percentage of anaphylaxis is more relevant, the use of adrenaline depends on factors such as the irregular availability of adrenaline auto-injectors in the world^47^ and the level of education of patients in the use of the drug, which is likely related to the availability of specific action plans. In terms of application of the DEFASE score, however, the number of patients who will be assigned a score of 3 points in Domain B will be 1.3% of the food allergy sufferers, while the number of patients who will be assigned a score of 2 points in the same domain will be 4.9%. Here also we registered large regional variations. For the maximum score, the frequency ranges between none in Southern and South-Eastern Asia to 1.9% in Western Asia up to 2.5% in North America. For the 2 points score, the frequency ranges between 0.02% in Southern Asia to 16% in Southern Africa.

### Individual minimal eliciting dose

Our method is not suitable for generating information about the reactions to small doses of food in the community. In fact, the table we proposed is purely indicative, and has no relation to the protein content of foods.^13^ However, we observed that the proportion of patients in whom the reaction can be attributed to a low individual eliciting dose was 16.51%. This response does not appear to present variations between the different geographical contexts. The data indicated that the number of patients to whom the highest severity score in Domain C can be attributed was around 1 in 6.

### Current impact on food allergy-related quality-of-life (FA-QoL)

While food allergy-related quality-of-life assessment scales are now well structured, and validated in several languages,^48^ they are not in common use in clinical practice. For this reason, we were not able to request numerical evaluations from the centers covered by the survey, but we asked more generally which percentage of patients see their quality of their minimally, moderately or severely influenced by food allergy. Not surprisingly, this is the item that, if translated to the DEFASE grid, contributes the most to the severity scale of the food allergy. Approximately 36.8% of patients may receive a score of 3 in this domain, with an impact described as significant. In several regions, the quality of life is described severely affected by more than 40% of patients. Among these, Eastern Europe (50%), Central and South America (45%), South Asia (43.3%), Western Asia (42.4%), and Southern Africa (40%) The Japanese exception suggests how local situations, such as the culture of individuals, families and the population, an appropriate food allergen labeling and the presence of a legislation on precautionary labeling, can have a favorable impact on this aspect.

### Current health-economic impact

It is not possible to estimate the total expenses that a typical patient with food allergies will have to face each year simply based on of Table 7. These will be far different depending on the need of the severity of the condition, the number of reactions that the patient presents, and the need for more or less frequent diagnostic evaluations. However, we can predict with certainty that the costs will be far different in the different regions of the world.

For the purposes of this discussion, we will refer to a typical patient who is followed for food allergies and has sporadic reactions (scenario A), and to another food-allergic patient who instead presents a severe reaction requiring hospitalization in an intensive care unit (scenario B).

In scenario A, we will include the yearly cost of 2 doses of adrenaline, 1 visit without a subsequent stay in the emergency room, 3 allergy visits, a dietary visit, a battery of diagnostic procedures including skin testing, molecular diagnostics, specific IgE and 1 oral provocation tests, 2 visits to the general practitioner, and 1 visit to the psychologist. According to our data, this patient is expected to incur an annual expense ranging between $481 in North Africa and $5335 in North America. If the patient is a child, the expense will vary between $564 in South Asia and $5366 in North America. The average cost will be $1689 and $1707, respectively.

In scenario B, we will include the same patient when she presents a reaction during the year requiring not only a visit, but also observation in the emergency room for 6 h; and then admission to an intensive care unit. In this case, the cost per year will range between $985 and $11,332 for adults, and between $990 and $11,363 for a pediatric patient, in Western Europe and North America respectively. The average yearly cost will be $2486 for adult patients and $2506 for pediatric patients.

In any case, the economic impact will be differently perceived and objectively different in different regions of the world depending on local economic conditions. In this regard, the calculation of the ratio between expenses for food allergy and gross national income per capita, as reported by the United Nations Trade and Development (UNCTAD) Organization,^49^ may be indicative (Table S11). A patient who experiences a reaction during the year without admission to intensive care can expect to spend between 1.4% in Western Europe and 38.4% in Southern Africa of their average annual income to manage the situation. An admission to intensive care due to food allergies is prohibitive in Southern Africa where it would cost 92.6% of the average per capita income, and more affordable in other regions such as Western Europe where it would account for 2.1% of the per capita income. Globally, a patient with food allergy can expect to spend between 5.9% in scenario A and 11.8% in scenario B of their income to manage their disease.

### Strengths and limitations

This study has some strengths and several limitations.^50^ Among the first, a clear definition of the research question and the associated hypotheses and the ability to capture information otherwise difficult to obtain. The questionnaire was reviewed by an expert panel, ensuring that the content and scope adequately address the research question.

Our target population is based on the WAO database, a unique source for obtaining opinions of professionals in the allergy sector. As many surveys, the FASE questionnaire is based on a report of opinions; the professionalism of their source guarantees their accuracy. The survey was presented with a tailored introduction stating its goals and purposes (Supplementary appendix) and boosted to increase participation.

As mentioned above, our study design prevents us from presenting these data as reflecting the epidemiology of food allergy in different countries of the world. They should be framed in terms of reported frequency of patients presenting to the responding centers with these allergies.

Our survey presents a sampling bias, linked to the low number of responses obtained. The majority of respondents are centers specialized in food allergy. Since the sampling bias can impact the representativeness, validity and generalizability of the data, we could overestimate the frequency and severity of food allergy. Therefore, these data must be read with the awareness that they refer to the population of allergy sufferers followed in specialized centers and not to the general population. Another limitation was the necessary approximation of some calculation measures, such as those related to the individual minimum elicitation dose. Despite a quite large representatively, missing data from Central Asia and the Pacific Islands suggests caution In terms of global generalizability. Given the study design, we were unable to explore the influence of ethnic, cultural, and healthcare system differences in detail.

## Conclusions

The FASE survey highlights many needs for research developments in food allergy recognition, education, management and perception. We must and can do a lot to overcome the barriers indicated as part of difficult-to-treat allergies.

There are significant research needs in the evaluation of pharmaco-economics of food allergy. The most striking is the need for epidemiological work on food allergy: national data are reported only by a few centers. Knowing the difficulty of carrying out these studies and the various levels of epidemiology to which they can be traced,^51^^,^^52^ we think that this is a commitment that must be taken on by the world scientific community in the near future.

Using the FASE data to estimate the real-life performance of the DEFASE grid, we can estimate that 36% of patients will receive a score of 3 in the QoL Domain D, 16.5% in the “minimum eliciting dose” Domain C, but only 2.8% in the symptoms and signs Domain A and 1.3% in the therapeutic Domain B. It is impossible to say from our data what percentage of patients will end up receiving a score of 3 in the health/economic Domain E, however, even considering a high percentage of high scores in this domain, we might expect the percentage of patients receiving a diagnosis of high severity using the DEFASE grid to be restricted by Domains A and B around 3%. Although this is a rough conclusion, subject to verification with daily use of the grid, this percentage is similar to that of asthma patients who receive a diagnosis of severe refractory asthma.^53^, ^54^, ^55^ Therefore, the DEFASE tool can reflect the real situation. It is ready to be promoted for practical implementation.

## Informed consent

Not applicable.

## Availability of data and materials

All data supporting the findings are included in the manuscript or supplementary materials.

## Author contributions

Members of the Food Allergy Severity (FASE) Working Group: Stefania Arasi, Alessandro Fiocchi, Lamia Dahdah, Mario Morais-Almeida, Bryan L. Martin, Gary Wing-Kin Wong, Motohiro Ebisawa, Ignacio J Ansotegui. All authors contributed to data interpretation and manuscript revision. AF drafted the initial version. CW, MS, UN, and NWA deeply revised and harmonized it. All authors approved the final version of the manuscript.

## Ethics approval

Not applicable.

## Consent for publication

All authors approved the final version and its submission.

## Declaration of generative AI and AI-assisted technologies in the writing process

Nothing to disclose.

## Funding

Not applicable.

## Declarations of competing interest

AF has received Speaker honoraria and advisory panel consultancy outside the submitted work for Nutricia, Abbott, Danone, Stallergenes, DBV, Novartis. Funded research (Institution) from Sanofi, Novartis, Ferrero, DBV, GSK, Astrazeneca, Hipp GmBDH, Humana SpA.

IJA reports personal fees from Bayer, Bial, Cipla, Eurodrug, Faes Farma, Gebro, Glenmark, Opella, Menarini, MSD, Roxall and Sanofi outside the submitted work.

SA declares that she has participated as an advisory board member, and/or consultant, and/or speaker/chair at scientific meetings for Aimmune, DBV, Ferrero, Mabylon, Novartis, Stallergenes Greer, Thermo Fisher Scientific and Ulrich outside the submitted work. Funded research (Institution) from Italian Minister of Health and Italian Minister of Education.

BM declares Speaker honorarium and advisory panel consultancy from Sanofi and Merck.

ME reports personal fees from Viatris and SAB of ARS-Pharmaceuticals, Novartis, and Sanofi outside the submitted work.

PGB declares speaker honoraria from Novartis.

NGP reports that he has served as a consultant for, carried out clinical research with, received grants from, and/or has received honoraria from: Abbott Nutrition, Berlin – Chemie AG, Danone, DBV Technologies, GSK, HAL Allergy, Hyproca Nutrition, MED Maps SRL, Menarini, Nestle Nutrition Institute, Numil, Regneron, Vianex, Vibrant America MMA reports personal fees from Astrazeneca, Bayer, Eurodrugs, FAES Farma, GSK, Menarini, MSD, OM Pharma, Sanofi and Viatris, outside the submitted work.

All other authors have no conflict of interest within the scope of the submitted work.
