# Supplementary material for: Food allergy severity across the world: A World Allergy Organization international survey
Source: World Allergy Organ J. 2025 Nov 12;18(11):101123. doi: 10.1016/j.waojou.2025.101123 (PMC12657590; doi:10.1016/j.waojou.2025.101123)
Supplement: Multimedia component 1 [file mmc1.doc]

Food Allergy SEverity international survey

(The **FASE** Project).

Supplementary appendix

**The FASE questionnaire.**

Throughout this questionnaire, Food Allergen Severity is abbreviated to FASE and is described according to the last DEFASE metanalysis[[1]](#endnote-2).

All responses will be anonymized before its analysis and publication.

Please, bear in mind that “your Centre” – refers to the place where you provide food allergy diagnosis and treatment, whether independently in your own clinic or as a team within a hospital. In addition, “your patients” refer to both children and adults you may be diagnosing and treating.

This questionnaire will take from 15 to 25 minutes to complete, depending on your options.

**Section 1 - RESPONDER/CENTRE INFORMATION**

**Questions marked with * are mandatory**

**1. Surname**

**2. Name**

*** 3. E-Mail**

*** 4. Centre name**

*** 5. City where the center is located**

*** 6. Country of practice (for the center named above)**

*** 7. Which is the health care system in your country? (more than one option is allowed)**

⃝ Universal government-funded health system (single-payer healthcare)

⃝ Universal public insurance system

⃝ Universal public-private insurance system

⃝ Universal private health insurance system

⃝ Non-universal insurance system

**8. Does your center provide food allergy care within the public or private sector?**

⃝ Public practice

⃝ Private practice

⃝ Both

*** 9. As part of your practice, do your center offer..**

⃝ skin prick tests

⃝ total IgE determination

⃝ specific IgE determination

⃝ specific molecular IgE determination

⃝ diagnostic oral food challenges

⃝ food-Allergen Immunotherapy

⃝ treatment with biologic drugs:

- Omalizumab

- Dupilumab

- Mepolizumab

- Reslizumab

- Benralizumab

- Other (please specify) ……………………………………………….

***10. To which specialty do you belong? (more than one can be selected)**

⃝ Allergy

⃝ Pediatrics / pediatric allergy

⃝ Pulmonology / respiratory

⃝ Dermatology

⃝ ENT (Ear, Nose and Throat)

⃝ Immunology

⃝ Gastroenterology

⃝ General/Community Practitioner

⃝ Internal Medicine

⃝ Other (please specify) ……………………

**11. Are you interested in contributing to future WAO projects on FASE?**

If you are interested, your personal information will be included in a database to be used in future collaboration projects on DEFASE within WAO.

⃝ No, I´m not interested in further contacts

⃝ Yes, I´m willing to be contacted in the future for DEFASE - related projects

⃝ I accept to be cited as contributor in the acknowledgments section of the manuscript related to this survey

**Section 2 – FOOD ALLERGIC PATIENTS IN YOUR CENTRE**

**12. How many food allergic patients attend your center per year?**

Total number of patients …..

*** 13. Which foods are your patients allergic to? Indicate the percentage; given the possibility of multiple food allergy, the total can exceed 100)**

⃝ Milk

⃝ Egg

⃝ Peanut

⃝ Wheat

⃝ Hazelnut

⃝ Peach

⃝ nPru p 3

⃝ Fish

⃝ Seafood

⃝ Multiple food allergy

⃝ Other (please specify)…………………

*** 14. Which symptoms experience your food-allergic patients? Indicate the percentage (given the possibility of multiple symptoms, the total can exceed 100)**

⃝ Food-induced Oral Allergy Syndrome

⃝ Food-allergic rhinitis

⃝ Food-allergic asthma

⃝ Food-allergic atopic dermatitis

⃝ Food-allergic immediate urticaria

⃝ Food-induced anaphylaxis*

*anaphylaxis is defined as a serious systemic hypersensitivity reaction that is usually rapid in onset and may cause death. Severe anaphylaxis is characterized by potentially life-threatening compromise in airway, breathing and/or the circulation, and may occur without typical skin features or circulatory shock being present

*** 15. Which percentage of your anaphylactic patients exhibits difficult to manage issues:**

a. Failure to define triggering food allergens …. %

b. Failure to educate patient and family …. %

c. Failure to be prepared to manage reactions (e.g. no management plan or therapy) …. %

d. Failure to avoid the triggering allergen(s) …. %

e. Failure to properly treat a reaction …. %

*** 16. in the most severe previous food-allergic reaction, how many of your patients did experience… (the total cannot exceed 100)*:**

a. Only local cutaneous, mild-moderate gastrointestinal or rhinoconjunctivitis symptoms ⃝

b. Lower respiratory, laryngeal or cardiovascular symptoms or signs (“i.e. anaphylaxis”) ⃝

c. Anaphylaxis causing respiratory or circulatory failure ⃝

*** 17. The treatment of the most severe previous food-allergic reaction did require … (the total cannot exceed 100)*:**

a. antihistamines or corticosteroids only ⃝

b. maximum 2 doses of i.m. adrenaline ⃝

c. Three of more doses of i.m. adrenaline, or an adrenaline infusion ⃝

*** 18. In your clinical experience, the food-triggering dose in the most severe food-allergic reaction of your patients was…**

a. Half of an age-appropriate portion of food in ……% patients

b. Less than half of an age appropriate portion of food in ……% patients

c. A very small amount dose in ……% patients[[2]](#endnote-3)

**19. What % of patients experience more than one severe reaction*** **per in the last 12 months?**

….…. %

*Sever reaction is defined as a reaction reported with two or more ‘red’ criteria in the overview of all anaphylaxis symptom-severity scores for listed symptom by organ, Figure S1[[3]](#endnote-4)

**20. It is known that the Quality of Life (QoL) of food allergy sufferers may be affected by dietary restrictions, emotional impact, risk of accidental exposure, social and dietary limitations. Which percentage of your patients displays (the total cannot exceed 100)*:**

a. No/minimal impact on QoL ….…. %

b. Moderate impact on QoL ….…. %

c. Significant impact on QoL ….…. %

**21. Which is the current currency in your country?**

**22. For each of the following expenses, please provide the current average cost in your currency**

a. One outpatient visit to the allergy specialist ________

b. One outpatient visit to the dietician ________

c. One outpatient visits to the psychologist ________

d. One community visit to general practitioner ________

e. One community visit to general pediatrician ________

f. serum test panel (7 extracts) ________

g. molecular diagnostic test (12 allergenic molecules)

h. cutaneous test (7 extracts) ________

i. one oral food challenge ________

j. one emergency department visit ________

k. one emergency department admission ________

l. emergency ambulance call ________

m. one day spent in ICU because of food allergy ________

n. one adrenaline(/epinephrine) auto-injector ________

**23. the health-economic status (HES) of food allergy sufferers may be affected by costs for outpatient visit due to food allergy, travel, ED admission, ambulance call(s), time spent in ICU, self-injectable epinephrine, loss of schooldays, loss of workdays, special foods, special school, special holidays, additional health insurance. Which percentage of your patients displays (the total cannot exceed 100)*:**

a. No/minimal impact on HES ….…. %

b. Moderate impact on HES ….…. %

c. Significant impact on HES ….…. %

**Section 3 – FOOD ALLERGIC PATIENTS IN YOUR COUNTRY**

**24. In the last 10 years, has food allergy in your country increased, decreased, or remained stable? (If no published data, please provide estimate based on any changes in clinical service burden or health care service activity)**

⃝ Increased

⃝ Decreased

⃝ Remained Stable

**This is based on:**

⃝ Clinical service burden (i.e change in patients referred / seen)

⃝ Published evidence. If published data, give reference:

**25. In the last 10 years, has food allergy severity in children of your country increased, decreased, or remained stable? (If no published data, please provide estimate based on any changes in clinical service burden or health care service activity)**

⃝ Increased

⃝ Decreased

⃝ Remained Stable

This is based on:

⃝ Clinical service burden (i.e change in patients referred / seen)

⃝ Published evidence. If published data, give reference:

**26. If there has been an increase in food allergy severity in the last 10 years, in which age group has this increase been seen mostly? (If no published data, please provide estimate based on any changes in clinical service burden or health care service activity)**

⃝ Infants < 1 year

⃝ 1-5 year olds

⃝ 6 – 12 year olds

⃝ 12 – 18 year olds

⃝ adults

**27. Is there published information available regarding the overall prevalence of food allergy in your country?**

⃝ Yes

⃝ No

⃝ Unsure

If published data, please provide reference: …………………………………………

**28. Based on any data available, what is the estimated percentage of patients in your country’s population who have food allergy (give any data available)**

⃝ Total % patients with food allergy ….. ％

⃝ Children < 5years ….. ％

⃝ Children > 5years ….. ％

⃝ 6 – 12 year olds ….. ％

⃝ 12 – 18 year olds ….. ％

⃝ adults ….. ％

**29. The answer above is based on:**

⃝ Estimated percentage

⃝ No answer was given because there is insufficient data to provide estimate

⃝ Published percentage (give reference):

**30. What are the most common clinical presentations of food allergy in children of your country (excluding food intolerances with no immune basis)?**

Please indicate list in decreasing order of frequency (If no published data, please estimate)

% Acute IgE-mediated food allergy (i.e. onset generally within 1-2 hours with angioedema, urticaria, vomiting or anaphylaxis) ⃝

% Non IgE-mediated food allergies (i.e. more delayed gastrointestinal symptoms ONLY e.g. reflux, constipation, colic, chronic diarrhea, blood in stool, WITHOUT IgE associated symptoms) ⃝

% Mixed IgE and non-IgE food allergy (children with features of both acute onset symptoms and more chronic symptoms, such as food allergy, exacerbated by foods) ⃝

**31. The answer above is based on:**

⃝ Estimated percentage

⃝ No answer was given because there is insufficient data to provide estimate

⃝ Published percentage (give references):

**32. What are the 5 most common FOOD ALLERGENS TRIGGERS in YOUNG children LESS THAN 5 years in your country?**

**1**

**2**

**3**

**4**

**5**

**33. The answer above is based on:**

⃝ Estimated percentage

⃝ No answer was given because there is insufficient data to provide estimate

⃝ Published percentage (give references):

**34. What are the five most common food allergens triggers in older children (5 – 11 years) in your country? (Please list allergens in order of prevalence):**

**1**

**2**

**3**

**4**

**5**

**35. The answer above is based on:**

⃝ Estimated percentage

⃝ No answer was given because there is insufficient data to provide estimate

⃝ Published percentage (give references):

**36. What are the five most common food allergens triggers in adolescents (12 – 18 years) in your country? (Please list allergens in order of prevalence):**

**1**

**2**

**3**

**4**

**5**

**37. The answer above is based on:**

⃝ Estimated percentage

⃝ No answer was given because there is insufficient data to provide estimate

⃝ Published percentage (give references):

**38. What are the five most common food allergens triggers in adults (18 years and older) in your country? (Please list allergens in order of prevalence):**

**1**

**2**

**3**

**4**

**5**

**39. The answer above is based on:**

⃝ Estimated percentage

⃝ No answer was given because there is insufficient data to provide estimate

⃝ Published percentage (give references):

**40. Do you have standardized National Anaphylaxis Action Plans (i.e are patients with a risk of anaphylaxis because of known allergy in your country given the same standardized management plan?)**

⃝ Yes

⃝ No

**If NO, which of the following apply (chose any/all that are relevant)**

⃝ Individual physicians provide Anaphylaxis Action Plans of their own choosing

⃝ Your society recommends use of published Anaphylaxis Action Plans from other societies/countries

Please indicate which:

**＿＿＿＿＿＿＿＿＿＿＿＿＿＿＿＿＿＿＿＿＿＿＿＿＿＿＿＿＿＿**

**41. Are Adrenaline auto-injectors readily available for treatment of anaphylaxis in your region?**

⃝ Yes

⃝ No

**42. If YES, are Adrenaline auto-injectors subsidized by your health care system?**

⃝ Yes

⃝ No

**Figure S1** – Overview of all anaphylaxis symptom-severity scores for each listed symptom ordered by organ

**
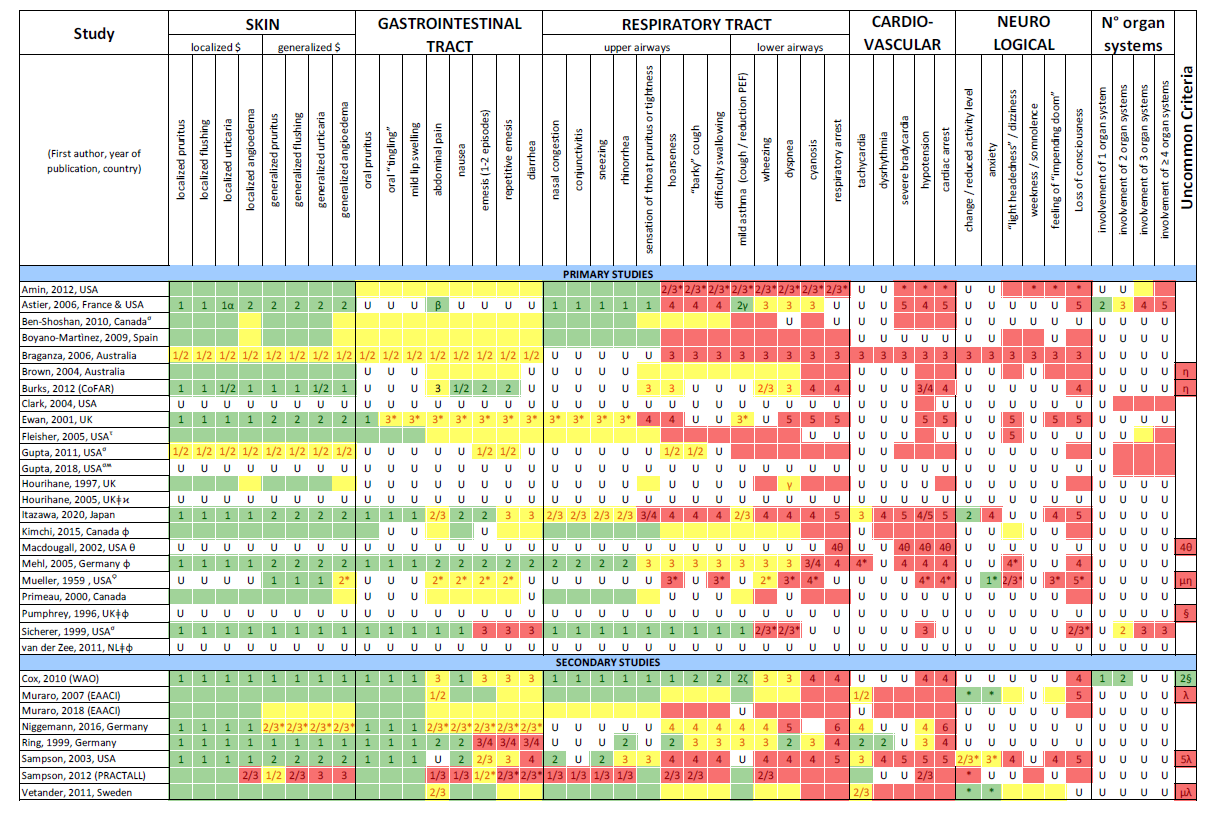
**

**Figure S2** – Difficult to treat food allergies, preliminary questions **
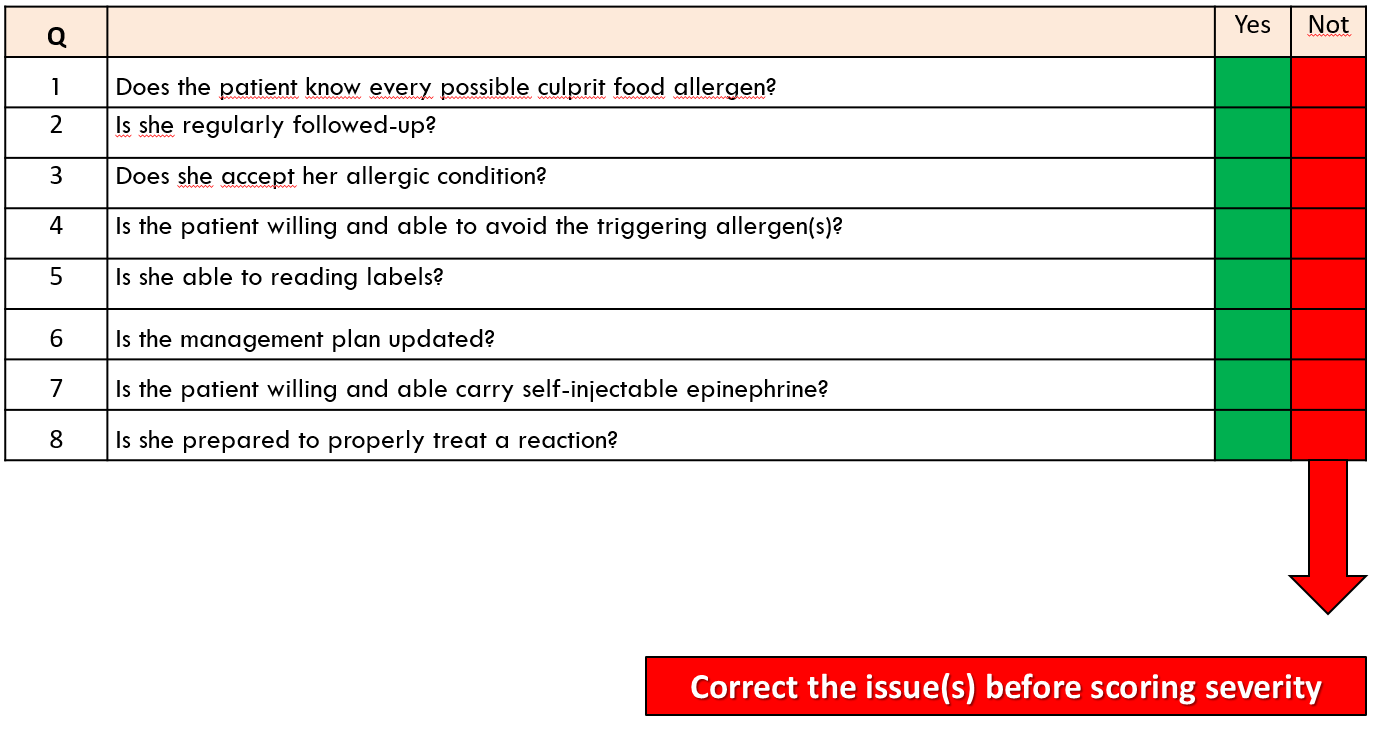
**

**Figure S3** – The DEFASE grid for food allergy severity.

**
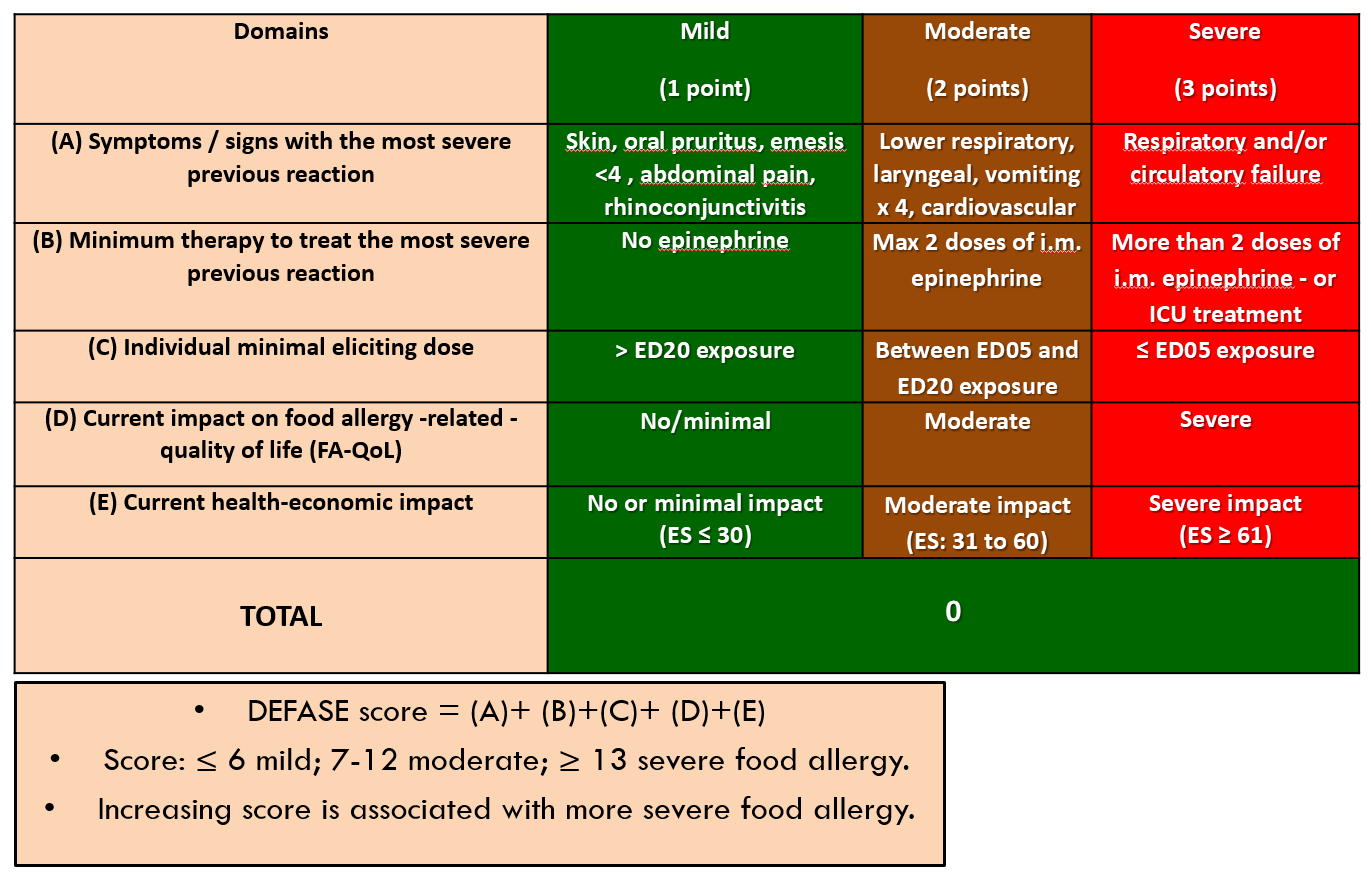
**

**Figure S4** – The DEFASE grid for wealth/health-economic impact


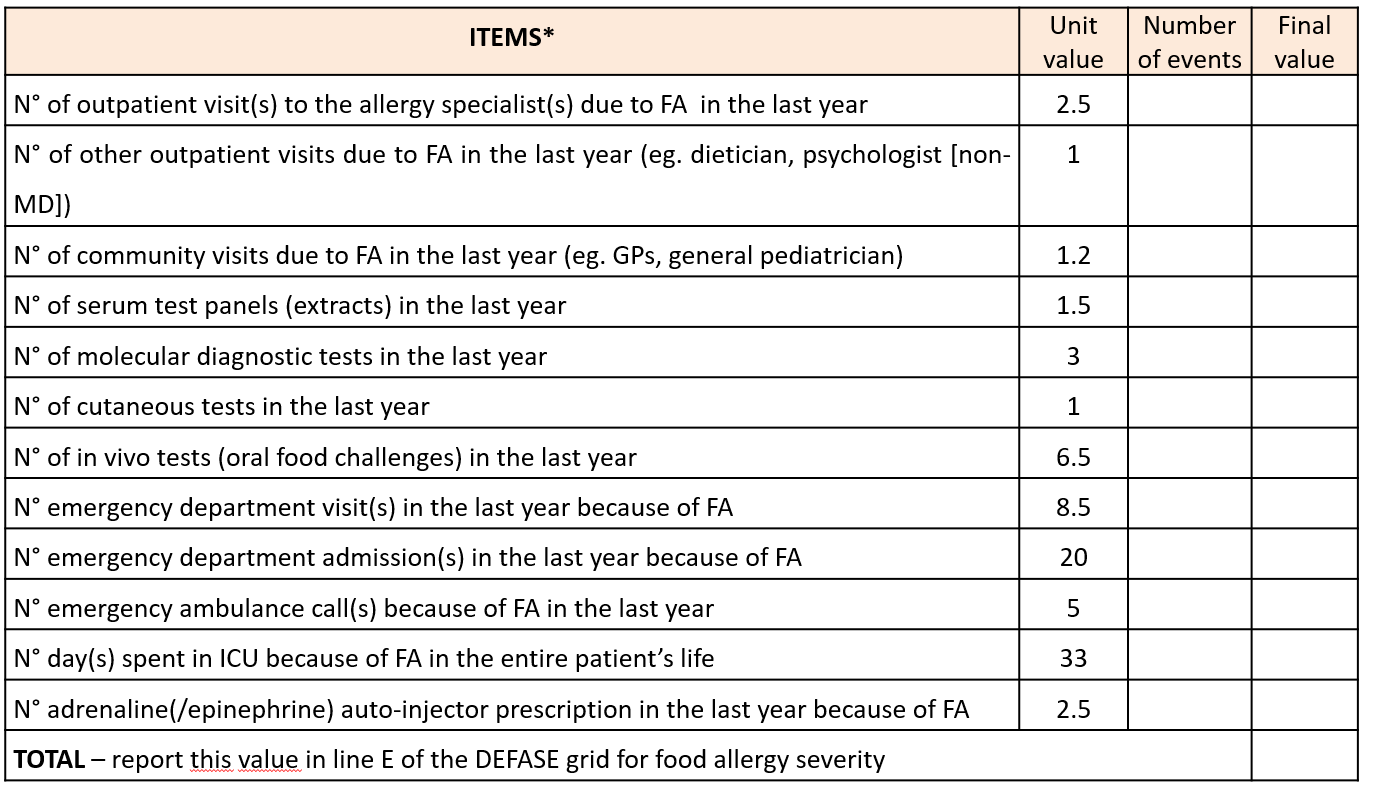


T**able S1 -** Geographical breakdown of the responding centers by country

**
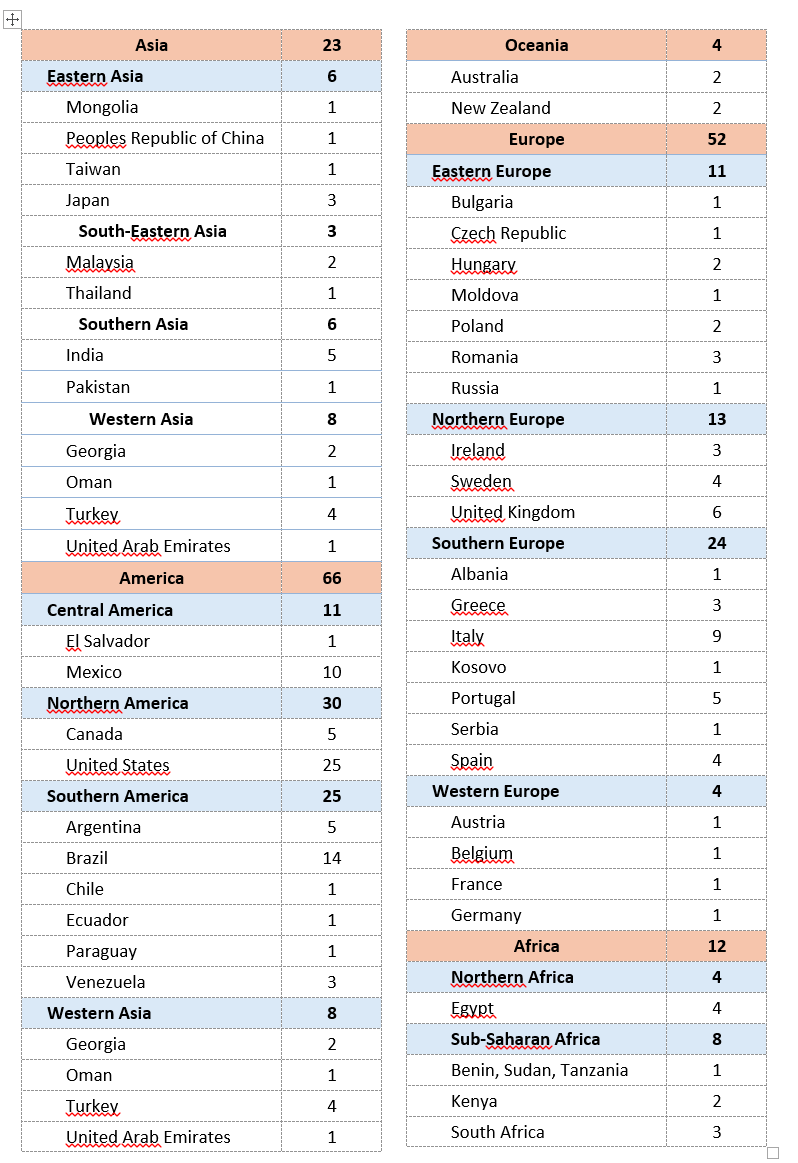
**

**TABLE S2 –** Percentage of patients reported with various manifestations of anaphylaxis in the most severe previous food-allergic reaction by the responding centres

| **Symptoms** | **Mean** |
| --- | --- |
| **Lower respiratory. laryngeal or cardiovascular symptoms or signs (“i.e. anaphylaxis”)** |  |
| Central America | 16.86 |
| Eastern Asia | 20.00 |
| Eastern Europe | 29.00 |
| Northern Africa | 29.67 |
| Northern America | 28.06 |
| Northern Europe | 13.40 |
| Oceania | 27.50 |
| South-Eastern Asia | 6.50 |
| Southern America | 25.56 |
| Southern Asia | 25.00 |
| Southern Europe | 31.17 |
| Sub-Saharan Africa | 43.00 |
| Western Asia | 24.43 |
| Western Europe | 30.00 |
| **Anaphylaxis causing respiratory or circulatory failure** |  |
| Central America | 4.86 |
| Eastern Asia | 10.50 |
| Eastern Europe | 12.20 |
| Northern Africa | 10.33 |
| Northern America | 11.12 |
| Northern Europe | 19.50 |
| Oceania | 7.50 |
| South-Eastern Asia | 6.00 |
| Southern America | 13.82 |
| Southern Asia | 5.00 |
| Southern Europe | 7.47 |
| Sub-Saharan Africa | 7.00 |
| Western Asia | 31.40 |
| Western Europe | 13.00 |
| **Only local cutaneous. mild-moderate gastrointestinal or rhinoconjunctivitis symptoms** |  |
| Central America | 78.29 |
| Eastern Asia | 69.50 |
| Eastern Europe | 65.67 |
| Northern Africa | 60.00 |
| Northern America | 65.06 |
| Northern Europe | 83.20 |
| Oceania | 65.00 |
| South-Eastern Asia | 87.50 |
| Southern America | 61.39 |
| Southern Asia | 70.00 |
| Southern Europe | 61.78 |
| Sub-Saharan Africa | 50.00 |
| Western Asia | 53.14 |
| Western Europe | 57.00 |
| **Lower respiratory. laryngeal or cardiovascular symptoms or signs (“i.e. anaphylaxis”) overall** | **26.19** |
| **Anaphylaxis causing respiratory or circulatory failure overall** | **11.66** |
| **Only local cutaneous. mild-moderate gastrointestinal or rhinoconjunctivitis symptoms overall** | **64.75** |

**TABLE S3 –** Type of treatment needed by the patients reported with anaphylaxis in the most severe previous food-allergic reaction, as reported by the responding centres

| **Treatment** | **Mean** |
| --- | --- |
| **No treatment** |  |
| Central America | 25.96 |
| Eastern Asia | 5.00 |
| Eastern Europe | 2.00 |
| Northern Africa | 23.33 |
| Northern America | 21.20 |
| Northern Europe | 37.83 |
| Oceania | 20.00 |
| South-Eastern Asia | 70.00 |
| Southern America | 20.72 |
| Southern Asia | 31.67 |
| Southern Europe | 32.89 |
| Sub-Saharan Africa | 33.33 |
| Western Asia | 19.62 |
| Western Europe | 35.00 |
| **Antihistamines or corticosteroids only** |  |
| Central America | 49.57 |
| Eastern Asia | 47.50 |
| Eastern Europe | 71.33 |
| Northern Africa | 63.17 |
| Northern America | 39.13 |
| Northern Europe | 48.50 |
| Oceania | 52.50 |
| South-Eastern Asia | 17.50 |
| Southern America | 54.31 |
| Southern Asia | 65.00 |
| Southern Europe | 45.50 |
| Sub-Saharan Africa | 10.42 |
| Western Asia | 30.32 |
| Western Europe | 32.50 |
| **Maximum 2 doses of i.m. adrenaline** |  |
| Central America | 19.14 |
| Eastern Asia | 40.00 |
| Eastern Europe | 16.00 |
| Northern Africa | 13.00 |
| Northern America | 34.50 |
| Northern Europe | 10.00 |
| Oceania | 24.50 |
| South-Eastern Asia | 12.50 |
| Southern America | 22.47 |
| Southern Asia | 3.33 |
| Southern Europe | 19.11 |
| Sub-Saharan Africa | 50.00 |
| Western Asia | 38.55 |
| Western Europe | 27.00 |
| **Three of more doses of i.m. adrenaline. or an adrenaline infusion** |  |
| Central America | 5.33 |
| Eastern Asia | 7.50 |
| Eastern Europe | 10.67 |
| Northern Africa | 0.50 |
| Northern America | 5.17 |
| Northern Europe | 3.67 |
| Oceania | 3.00 |
| South-Eastern Asia | 0 |
| Southern America | 2.50 |
| Southern Asia | 0 |
| Southern Europe | 2.50 |
| Sub-Saharan Africa | 6.25 |
| Western Asia | 11.50 |
| Western Europe | 5.50 |
| **No treatment (overall)** | **27.66** |
| **Antihistamines or corticosteroids only (overall)** | **49.60** |
| **Maximum 2 doses of i.m. adrenaline (overall)** | **24.35** |
| **Three of more doses of i.m. adrenaline. or an adrenaline infusion (overall)** | **5.65** |

**TABLE S4 –** Food-triggering dose in the most severe previous food-allergic reaction, as reported by the responding centres.

| **Food triggering dose** | **Mean** |
| --- | --- |
| **The whole age-appropriate portion of food in (%) patients** |  |
| Central America | 37.00 |
| Eastern Asia | 0 |
| Eastern Europe | 6.67 |
| Northern Africa | 65.00 |
| Northern America | 10.27 |
| Northern Europe | 38.75 |
| Oceania | 45.00 |
| South-Eastern Asia | 50.00 |
| Southern America | 20.91 |
| Southern Asia | 22.50 |
| Southern Europe | 35.13 |
| Sub-Saharan Africa | 30.00 |
| Western Asia | 47.00 |
| Western Europe | 25.00 |
| **Half of an age-appropriate portion of food in (%) patients** |  |
| Central America | 23.00 |
| Eastern Asia | 5.00 |
| Eastern Europe | 30.00 |
| Northern Africa | 25.00 |
| Northern America | 18.23 |
| Northern Europe | 25.50 |
| Oceania | 10.00 |
| South-Eastern Asia | 40.00 |
| Southern America | 31.67 |
| Southern Asia | 40.00 |
| Southern Europe | 23.67 |
| Sub-Saharan Africa | 37.50 |
| Western Asia | 22.50 |
| Western Europe | 25.00 |
| **Less than half of an age appropriate portion of food in (%) patients** |  |
| Central America | 27.50 |
| Eastern Asia | 5.00 |
| Eastern Europe | 22.33 |
| Northern Africa | 8.00 |
| Northern America | 56.15 |
| Northern Europe | 22.33 |
| Oceania | 40.00 |
| South-Eastern Asia | 10.00 |
| Southern America | 30.83 |
| Southern Asia | 37.50 |
| Southern Europe | 24.25 |
| Sub-Saharan Africa | 32.50 |
| Western Asia | 17.25 |
| Western Europe | 40.00 |
| **A very small amount dose in (%) patients** |  |
| Central America | 12.5 |
| Eastern Asia | 90.00 |
| Eastern Europe | 41.00 |
| Northern Africa | 2.00 |
| Northern America | 15.35 |
| Northern Europe | 13.42 |
| Oceania | 5.00 |
| South-Eastern Asia | -0 |
| Southern America | 11.59 |
| Southern Asia | 0 |
| Southern Europe | 16.95 |
| Sub-Saharan Africa | 0 |
| Western Asia | 13.25 |
| Western Europe | 10.00 |
| **The whole age-appropriate portion of food (overall)** | **31.66** |
| **Half of an age-appropriate portion of food (overall)** | **25.14** |
| **Less than half of an age appropriate portion of food (overall)** | **26.69** |
| **A very small amount dose(overall)** | **16.51** |

**TABLE S5 –** Estimated impact of dietary restrictions, social and dietary limitations on quality of life of food allergy sufferers.

| **Impact on QoL in (%) patients, mean by world region** | | | |
| --- | --- | --- | --- |
|  | **Significant** | **Moderate** | **No/minimal** |
| Central America | 45.00 | 35.71 | 19.29 |
| Eastern Asia | 1.00 | 19.00 | 80.00 |
| Eastern Europe | 50.00 | 34.00 | 16.00 |
| Northern Africa | 30.00 | 30.00 | 40.00 |
| Northern America | 33.47 | 49.87 | 17.86 |
| Northern Europe | 15.83 | 31.67 | 52.50 |
| Oceania | 20.00 | 65.00 | 15.00 |
| South-Eastern Asia | 15.00 | 30.00 | 55.00 |
| Southern America | 45.00 | 36.76 | 18.24 |
| Southern Asia | 43.33 | 45.00 | 11.67 |
| Southern Europe | 37.00 | 51.67 | 12.14 |
| Sub-Saharan Africa | 40.00 | 31.67 | 28.33 |
| Western Asia | 42.43 | 44.67 | 22.50 |
| Western Europe | 25.00 | 65.00 | 10.00 |
| **Significant impact on QoL (overall)** | **36.82** | | |
| **Moderate impact on QoL (overall)** | **42.36** | | |
| **No/minimal impact on QoL (overall)** | **22.06** | | |

**TABLE S6 – Prevalence of food-specific allergies in young children under 5 years of age, as reported by the responding centres**

| **1^st^ most common food trigger** | **N** | **%** |
| --- | --- | --- |
| milk | 57 | 77.03 |
| egg | 10 | 13.51 |
| peanut | 6 | 8.11 |
| fruit | 1 | 1.35 |
| **Overall** | **74** | **100** |

| **2^nd^ most common food trigger** | **N** | **%** |
| --- | --- | --- |
| egg | 53 | 72.60 |
| milk | 9 | 12.33 |
| peanut | 5 | 6.85 |
| wheat | 2 | 2.74 |
| fish | 2 | 2.74 |
| meat | 1 | 1.37 |
| soy | 1 | 1.37 |
| **Overall** | **73** | **100** |

| **3^rd^ most common food trigger** | **N** | **%** |
| --- | --- | --- |
| peanut | 13 | 18.31 |
| tree nut | 12 | 16.90 |
| wheat | 10 | 14.08 |
| fish | 9 | 12.68 |
| milk | 6 | 8.45 |
| soy | 6 | 8.45 |
| egg | 5 | 7.04 |
| shellfish | 5 | 7.04 |
| fruit | 2 | 2.82 |
| vegetables | 2 | 2.82 |
| oilseeds | 1 | 1.41 |
| **Overall** | **71** | **100** |

| **4^th^ most common food trigger** | **N** | **%** |
| --- | --- | --- |
| tree nut | 22 | 34.92 |
| peanut | 8 | 12.70 |
| fish | 6 | 9.52 |
| wheat | 6 | 9.52 |
| shellfish | 6 | 9.52 |
| fruit | 5 | 7.94 |
| oilseeds | 3 | 4.76 |
| soy | 2 | 3.17 |
| peach/LTP | 1 | 1.59 |
| egg | 1 | 1.59 |
| milk | 1 | 1.59 |
| additives | 1 | 1.59 |
| vegetables | 1 | 1.59 |
| **Overall** | **63** | **100** |

| **5^th^ most common food trigger** | **N** | **%** |
| --- | --- | --- |
| wheat | 12 | 21.05 |
| fish | 9 | 15.79 |
| tree nut | 8 | 14.04 |
| soy | 5 | 8.77 |
| oilseeds | 4 | 7.02 |
| shellfish | 4 | 7.02 |
| peanut | 4 | 7.02 |
| egg | 3 | 5.26 |
| fruit | 3 | 5.26 |
| peach/LTP | 1 | 1.75 |
| milk | 1 | 1.75 |
| additives | 1 | 1.75 |
| peanut | 1 | 1.75 |
| legumes | 1 | 1.75 |
| **Overall** | **57** | **100** |

**The answer above is based on:**

|  | **N=83** |
| --- | --- |
| Estimated percentage | 73.49% |
| No answer was given because there is insufficient data to provide estimate | 15.66% |
| Published percentage | 10.85% |

**TABLE S7 – Prevalence of food-specific allergies in children 5 – 11 years of age, as reported by the responding centres**

| **1^st^ most common food trigger** | **N** | **%** |
| --- | --- | --- |
| peanut | 20 | 28.99 |
| egg | 19 | 27.54 |
| milk | 16 | 23.19 |
| shellfish | 5 | 7.25 |
| tree nut | 4 | 5.80 |
| wheat | 2 | 2.90 |
| fruit | 2 | 2.90 |
| peanut | 1 | 1.45 |
| **Overall** | **69** | **100** |

| **2^nd^ most common food trigger** | **N** | **%** |
| --- | --- | --- |
| tree nut | 19 | 28.36 |
| egg | 16 | 23.88 |
| milk | 13 | 19.40 |
| fish | 6 | 8.96 |
| fruit | 3 | 4.48 |
| peanut | 3 | 4.48 |
| shellfish | 2 | 2.99 |
| fruit | 1 | 1.49 |
| meat | 1 | 1.49 |
| almond | 1 | 1.49 |
| corn | 1 | 1.49 |
| soy | 1 | 1.49 |
| **Overall** | **67** | **100** |

| **3^rd^ most common food trigger** | **N** | **%** |
| --- | --- | --- |
| shellfish | 10 | 15.15 |
| egg | 8 | 12.12 |
| wheat | 7 | 10.61 |
| tree nut | 7 | 10.61 |
| milk | 7 | 10.61 |
| peanut | 7 | 10.61 |
| fruit | 5 | 7.58 |
| oilseeds | 5 | 7.58 |
| soy | 4 | 6.06 |
| fish | 3 | 4.55 |
| vegetables | 1 | 1.52 |
| legumes | 1 | 1.52 |
| chocolate | 1 | 1.52 |
| **Overall** | **66** | **100** |

| **4^th^ most common food trigger** | **N** | **%** |
| --- | --- | --- |
| fish | 12 | 20.34 |
| milk | 9 | 15.25 |
| shellfish | 9 | 15.25 |
| egg | 7 | 11.86 |
| tree nut | 7 | 11.86 |
| fruit | 5 | 8.47 |
| peanut | 4 | 6.78 |
| peach/LTP | 3 | 5.08 |
| wheat | 2 | 3.39 |
| oilseeds | 1 | 1.69 |
| **Overall** | **59** | **100** |

| **5^th^ most common food trigger** | **N** | **%** |
| --- | --- | --- |
| shellfish | 13 | 25.49 |
| tree nut | 6 | 11.76 |
| wheat | 6 | 11.76 |
| fish | 5 | 9.80 |
| milk | 5 | 9.80 |
| oilseeds | 3 | 5.88 |
| peanut | 3 | 5.88 |
| egg | 3 | 5.88 |
| fruit | 2 | 3.92 |
| soy | 2 | 3.92 |
| peach/LTP | 1 | 1.96 |
| wheat | 1 | 1.96 |
| additives | 1 | 1.96 |
| **Overall** | **51** | **100** |

**The answer above is based on:**

|  | **N=83** |
| --- | --- |
| Estimated percentage | 69.87% |
| No answer was given because there is insufficient data to provide estimate | 20.48% |
| Published percentage | 9.69% |

**TABLE S8 – Prevalence of food-specific allergies in adolescents 12 – 18 years of age, as reported by the responding centres**

| **1^st^ most common food trigger** | **N** | **%** |
| --- | --- | --- |
| peanut | 26 | 40.00 |
| shellfish | 13 | 20.00 |
| tree nut | 10 | 15.38 |
| milk | 7 | 10.77 |
| fruit | 3 | 4.62 |
| peach/LTP | 2 | 3.08 |
| egg | 2 | 3.08 |
| fish | 1 | 1.54 |
| wheat | 1 | 1.54 |
| **Overall** | **65** | **100** |

| **2^nd^ most common food trigger** | **N** | **%** |
| --- | --- | --- |
| tree nut | 16 | 25.40 |
| shellfish | 11 | 17.46 |
| fish | 9 | 14.29 |
| peanut | 7 | 11.11 |
| fruit | 5 | 7.94 |
| egg | 4 | 6.35 |
| milk | 3 | 4.76 |
| wheat | 2 | 3.17 |
| peach/LTP | 1 | 1.59 |
| vegetable | 1 | 1.59 |
| beans | 1 | 1.59 |
| vegetables | 1 | 1.59 |
| meat | 1 | 1.59 |
| soy | 1 | 1.59 |
| **Overall** | **63** | **100** |

| **3^rd^ most common food trigger** | **N** | **%** |
| --- | --- | --- |
| tree nut | 11 | 18.03 |
| shellfish | 10 | 16.39 |
| fruit | 7 | 11.48 |
| egg | 6 | 9.84 |
| peanut | 6 | 9.84 |
| milk | 6 | 9.84 |
| oilseeds | 4 | 6.56 |
| wheat | 3 | 4.92 |
| fish | 3 | 4.92 |
| additives | 1 | 1.64 |
| meat | 1 | 1.64 |
| legumes | 1 | 1.64 |
| vegetables | 1 | 1.64 |
| soy | 1 | 1.64 |
| **Overall** | **61** | **100** |

| **4^th^ most common food trigger** | **N** | **%** |
| --- | --- | --- |
| fish | 12 | 23.53 |
| shellfish | 10 | 19.61 |
| egg | 7 | 13.73 |
| milk | 7 | 13.73 |
| tree nut | 4 | 7.84 |
| fruit | 3 | 5.88 |
| wheat | 2 | 3.92 |
| soy | 2 | 3.92 |
| peach/LTP | 1 | 1.96 |
| oilseeds | 1 | 1.96 |
| chocolate | 1 | 1.96 |
| peanut | 1 | 1.96 |
| **Overall** | **51** | **100** |

| **5^th^ most common food trigger** | **N** | **%** |
| --- | --- | --- |
| fish | 11 | 21.57 |
| shellfish | 11 | 21.57 |
| milk | 9 | 17.65 |
| egg | 5 | 9.80 |
| tree nut | 3 | 5.88 |
| oilseeds | 3 | 5.88 |
| wheat | 2 | 3.92 |
| fruit | 2 | 3.92 |
| soy | 2 | 3.92 |
| peach/LTP | 1 | 1.96 |
| peanut | 1 | 1.96 |
| meat | 1 | 1.96 |
| **Overall** | **51** | **100** |

**The answer above is based on:**

|  | **N=83** |
| --- | --- |
| Estimated percentage | 69.88% |
| No answer was given because there is insufficient data to provide estimate | 22.89% |
| Published percentage | 7.23% |

**TABLE S9 – Prevalence of food-specific allergies in adults over 18 years of age, as reported by the responding centres**

| **1^st^ most common food trigger** | **N** | **%** |
| --- | --- | --- |
| shellfish | 22 | 33.33 |
| peanut | 12 | 18.18 |
| fish | 7 | 10.61 |
| tree nut | 7 | 10.61 |
| fruit | 6 | 9.09 |
| milk | 3 | 4.55 |
| egg | 3 | 4.55 |
| peach/LTP | 2 | 3.03 |
| wheat | 2 | 3.03 |
| vegetables | 1 | 1.52 |
| rice | 1 | 1.52 |
| **Overall** | **66** | **100** |

| **2^nd^ most common food trigger** | **N** | **%** |
| --- | --- | --- |
| tree nut | 16 | 25.40 |
| shellfish | 11 | 17.46 |
| fish | 9 | 14.29 |
| peanut | 7 | 11.11 |
| fruit | 5 | 7.94 |
| egg | 4 | 6.35 |
| milk | 3 | 4.76 |
| wheat | 2 | 3.17 |
| peach/LTP | 1 | 1.59 |
| vegetable | 1 | 1.59 |
| legumes | 1 | 1.59 |
| vegetables | 1 | 1.59 |
| meat | 1 | 1.59 |
| soy | 1 | 1.59 |
| **Overall** | **63** | **100** |

| **3^rd^ most common food trigger** | **N** | **%** |
| --- | --- | --- |
| tree nut | 13 | 21.67 |
| peanut | 9 | 15.00 |
| shellfish | 8 | 13.33 |
| fruit | 7 | 11.67 |
| wheat | 3 | 5.00 |
| egg | 3 | 5.00 |
| milk | 3 | 5.00 |
| fish | 3 | 5.00 |
| peach/LTP | 2 | 3.33 |
| profillin | 2 | 3.33 |
| oilseeds | 2 | 3.33 |
| legumes | 1 | 1.67 |
| meat | 1 | 1.67 |
| vegetables | 1 | 1.67 |
| soy | 1 | 1.67 |
| spices | 1 | 1.67 |
| **Overall** | **60** | **100** |

| **4^th^ most common food trigger** | **N** | **%** |
| --- | --- | --- |
| fish | 8 | 16.33 |
| egg | 6 | 12.24 |
| soy | 6 | 12.24 |
| shellfish | 6 | 12.24 |
| peanut | 5 | 10.20 |
| fruit | 5 | 10.20 |
| tree nut | 5 | 10.20 |
| wheat | 4 | 8.16 |
| milk | 2 | 4.08 |
| vegetables | 1 | 2.04 |
| legumes | 1 | 2.04 |
| **Overall** | **49** | **100** |

| **5^th^ most common food trigger** | **N** | **%** |
| --- | --- | --- |
| shellfish | 10 | 22.22 |
| fish | 8 | 17.78 |
| milk | 6 | 13.33 |
| tree nut | 5 | 11.11 |
| wheat | 4 | 8.89 |
| fruit | 3 | 6.67 |
| egg | 2 | 4.44 |
| peach/LTP | 1 | 2.22 |
| oilseeds | 1 | 2.22 |
| meat | 1 | 2.22 |
| mustard | 1 | 2.22 |
| legumes | 1 | 2.22 |
| profillin | 1 | 2.22 |
| peanut | 1 | 2.22 |
| **Overall** | **45** | **100** |

The answer above is based on:

|  | **N=83** |
| --- | --- |
| Estimated percentage | 73.49% |
| No answer was given because there is insufficient data to provide estimate | 21.69% |
| Published percentage | 4.82% |

**TABLE S10 – Availability of have standardized National Anaphylaxis Action Plans as reported by the responding centres. (i.e are patients with a risk of anaphylaxis because of known allergy in a specific country given the same standardized management plan?)**

|  | **N** | **%** |
| --- | --- | --- |
| No | 47 | 55,9 |
| Yes | 37 | 44,1 |
| **Respondents** | **84** | **100** |

If NO, which of the following apply (chose any/all that are relevant)

|  | **N** | **%** |
| --- | --- | --- |
| Individual physicians provide Anaphylaxis Action Plans of their own choosing | 30 | 63,83 |
| Your society recommends use of published Anaphylaxis Action Plans from other societies/countries | 10 | 21,28 |
| Both | 7 | 14,89 |

**TABLE S11 –** Yearly costs of food allergy by region, compared to gross domestic product per capita of the respective region, as reported by the UN Trade and Development (UNCTAD) Organization[[4]](#endnote-5).

**Scenario A**: yearly cost of two doses of adrenaline, one visit without a subsequent stay in the emergency room, three allergy visits. a dietary visit, a battery of diagnostic procedures including skin testing, molecular diagnostics, specific IgE and one oral provocation tests, two visits to the general practitioner, and a visit to the psychologist. **Scenario B**: same plus observation in the emergency room for six hours; and then admission to an intensive care unit in one occasion during the year.

| **Region** | **GNI pro capita** | **Food allergy cost,**  **scenario A** | **Ratio** | **Food allergy cost,**  **scenario B** | **Ratio** |
| --- | --- | --- | --- | --- | --- |
| North America | 61840 | 5335.18 | 8.6 | 11332 | 18.3 |
| Central America | 7113 | 907.42 | 12.7 | 2796.2 | 39.3 |
| South America | 6526 | 1045.12 | 16.0 | 1652.7 | 25.3 |
| Northern Europe | 44882 | 809.01 | 1.8 | 2380.3 | 5.3 |
| Eastern Europe | 10445 | 528.3 | 5.1 | 701.95 | 6.7 |
| Southern Europe | 25320 | 1015.3 | 4.0 | 2255.3 | 8.9 |
| Western Europe | 46725 | 672.5 | 1.4 | 985 | 2.1 |
| Western Asia | 10530 | 2876.95 | 27.3 | 3339.6 | 31.7 |
| Eastern Asia | 13430 | 816.5 | 6.1 | 2380.8 | 17.7 |
| Southern Asia | 2207 | 556 | 25.2 | 1068 | 48.4 |
| South-Eastern Asia | 4362 | 764.1 | 17.5 | 1736.6 | 39.8 |
| Oceania | 38649 | 1166.55 | 3.0 | 2171.6 | 5.6 |
| North Africa | 3019 | 481 | 15.9 | 659.53 | 21.8 |
| Sub-Saharan Africa | 1457 | 560.05 | 38.4 | 1349.7 | 92.6 |
| Mean | 21158 | 1252.4 | 5.9 | 2486.7 | 11.8 |

**References**

1. Arasi S, Nurmatov U, Dunn-Galvin A, Daher S, Roberts G, Turner PJ, Shinder SB, Gupta R, Eigenmann P, Nowak-Wegrzyn A, Sánchez Borges MA, Ansotegui IJ, Fernandez-Rivas M, Petrou S, Tanno LK, Vazquez-Ortiz M, Vickery BP, Wong GW, Ebisawa M, Fiocchi A. Consensus on DEfinition of Food Allergy SEverity (DEFASE) an integrated mixed methods systematic review. World Allergy Organ J. 2021;14:100503 [↑](#endnote-ref-2)
2. Bird JA, Leonard S, Groetch M, Assa'ad A, Cianferoni A, Clark A, Crain M, Fausnight T, Fleischer D, Green T, Greenhawt M, Herbert L, Lanser BJ, Mikhail I, Mustafa S, Noone S, Parrish C, Varshney P, Vlieg-Boerstra B, Young MC, Sicherer S, Nowak-Wegrzyn A. Conducting an Oral Food Challenge: An Update to the 2009 Adverse Reactions to Foods Committee Work Group Report. J Allergy Clin Immunol Pract. 2020;8:75-90.e17 [↑](#endnote-ref-3)
3. Arasi S, Nurmatov U, Turner PJ, Ansotegui IJ, Daher S, Dunn-Galvin A, Ebisawa M, Eigenmann P, Fernandez-Rivas M, Gupta R, Nowak-Wegrzyn A, Petrou S, Roberts G, Sánchez Borges MA, Sindher SB, Tanno LK, Vazquez-Ortiz M, Vickery BP, Wong GW, Fiocchi A. Consensus on DEfinition of Food Allergy SEverity (DEFASE): Protocol for a systematic review. World Allergy Organ J. 2020;13:100493 [↑](#endnote-ref-4)
4. <https://unctadstat.unctad.org/datacentre/dataviewer/US.GNI>, accessed July 20^th^, 2024. [↑](#endnote-ref-5)
